# Age Specific Survival Rates of Steller Sea Lions at Rookeries with Divergent Population Trends in the Russian Far East

**DOI:** 10.1371/journal.pone.0127292

**Published:** 2015-05-27

**Authors:** Alexey V. Altukhov, Russel D. Andrews, Donald G. Calkins, Thomas S. Gelatt, Eliezer D. Gurarie, Thomas R. Loughlin, Evgeny G. Mamaev, Victor S. Nikulin, Peter A. Permyakov, Sergey D. Ryazanov, Vladimir V. Vertyankin, Vladimir N. Burkanov

**Affiliations:** 1 University of Alaska Fairbanks, Fairbanks, Alaska, USA; 2 Kamchatka Branch of the Pacific Geographical Institute FEB RAS, Petropavlovsk-Kamchatsky, Kamchatsky Kray, Russia; 3 Alaska SeaLife Center, Seward, Alaska, USA; 4 North Pacific Wildlife Consulting LLC, Anchorage, Alaska, USA; 5 National Marine Mammal Laboratory (AFSC, NMFS, NOAA), Seattle, Washington, USA; 6 Department of Biology, University of Maryland, College Park, Maryland, USA; 7 School of Environmental and Forest Sciences, University of Washington, Seattle, Washington, USA; 8 TRL Wildlife Consulting (NMML retired), Seattle, Washington, USA; 9 State Nature Reserve “Komandorsky”, Nikolskoe, Kamchatsky Kray, Russia; 10 Kamchatka Research Institute of Fisheries and Oceanography, Petropavlovsk-Kamchatsky, Kamchatsky Kray, Russia; 11 V.I.Il‘ichev Pacific Oceanological Institute FEB RAS, Vladivostok, Primorsky Kray, Russia; 12 Kronotsky Reserve, Yelizovo, Kamchatsky Kray, Russia; National Cheng-Kung University, TAIWAN

## Abstract

After a dramatic population decline, Steller sea lions have begun to recover throughout most of their range. However, Steller sea lions in the Western Aleutians and Commander Islands are continuing to decline. Comparing survival rates between regions with different population trends may provide insights into the factors driving the dynamics, but published data on vital rates have been extremely scarce, especially in regions where the populations are still declining. Fortunately, an unprecedented dataset of marked Steller sea lions at rookeries in the Russian Far East is available, allowing us to determine age and sex specific survival in sea lions up to 22 years old. We focused on survival rates in three areas in the Russian range with differing population trends: the Commander Islands (Medny Island rookery), Eastern Kamchatka (Kozlov Cape rookery) and the Kuril Islands (four rookeries). Survival rates differed between these three regions, though not necessarily as predicted by population trends. Pup survival was higher where the populations were declining (Medny Island) or not recovering (Kozlov Cape) than in all Kuril Island rookeries. The lowest adult (> 3 years old) female survival was found on Medny Island and this may be responsible for the continued population decline there. However, the highest adult survival was found at Kozlov Cape, not in the Kuril Islands where the population is increasing, so we suggest that differences in birth rates might be an important driver of these divergent population trends. High pup survival on the Commander Islands and Kamchatka Coast may be a consequence of less frequent (e.g. biennial) reproduction there, which may permit females that skip birth years to invest more in their offspring, leading to higher pup survival, but this hypothesis awaits measurement of birth rates in these areas.

## Introduction

The global population of Steller sea lions declined dramatically, likely by over 75%, between the mid-1970’s through at least the late 1990’s [1–3]. By 1990, concern for the decline of the species prompted NOAA Fisheries to list the entire species as threatened under the U.S. Endangered Species Act (ESA). The Ministry of Natural Resources of Russia listed the species as endangered in the Red Data Book of Russia in 1994. By 1997 sufficient information was available to allow delineation of the population into two stocks, the western stock or western Distinct Population Segment (wDPS) consisting of Steller sea lions west of 144° W longitude, and an eastern stock or eDPS extending to the southern terminus of the range in central California [4]. Steller sea lions in the western DPS were elevated to “endangered” status under the U.S. ESA in 1997. More recent genetic studies suggest an additional stock or population division should separate the western Stock from an Asian stock west of 165° W longitude [5], consisting of rookeries in Kamchatka, the Kuril Islands, and the Sea of Okhotsk in eastern Russia. The single rookery on the Commander Islands, in far eastern Russia, groups genetically with the western stock in Alaska.

The Western DPS continued to decline throughout most of its range through the 1990’s, but by the year 2000 the decline had begun to slow and in most areas populations have been recovering recently [2]. The exception has been the small populations remaining in the Western and Central Aleutian Islands where numbers continue to fall [2]. Unfortunately, there are no demographic data other than population counts for Western Aleutian Islands Steller sea lions, and more generally, there is very little information on vital rates over the entire geographic range of Steller sea lions. Hastings et al. [6] recently provided vital rate estimations for southeastern Alaska Steller sea lions from the eastern DPS. They provided age specific estimates of survival, and found that early conditions (body mass at 1 month old and environmental conditions in the birth year) affected survival. Also, geographic conditions, such as local productivity, relative safety from predators, and local Steller sea lion population size were likely important determinants of age-specific survival rates. Horning and Mellish [7] also highlighted the role of contemporary predation rates, and using a density dependent conceptual framework they suggested that predation on juvenile sea lions could be the largest constraint on recovery of the species in the eastern Gulf of Alaska region. Their analysis highlighted the necessity for demographic models based on age-structured census data in order to incorporate the differential impact of predation on multiple vital rates [7]. Previous conclusions [8] that juvenile survival rates have recovered to pre-decline rates while natality has continued to fall, at least in the central Gulf of Alaska, have recently been questioned [7, 9]. In a recent analysis of Steller sea lion survival in the eastern, central, and western Gulf of Alaska, Fritz et al. [10] also noted that survival rates have rebounded to nearly the same levels estimated in the 1970’s prior to the population’s decline. Their population models indicate that current (2000–2012) natality of the increasing population east of Samalga Pass may not be significantly different from rates estimated for the 1970’s prior to the Western stock decline, but if they treat the CGOA as a closed population, natality is still lower than it was in the 1970’s [10]. However these studies were primarily based on data from the Gulf of Alaska during a period when that population was on the rise, so their results provide little insight into the current population dynamics in further west areas, especially in the Central and Western Aleutians where populations continue to decline. Unfortunately, there are currently no published data on vital rates for Steller sea lions west of Ugamak Island, at the eastern end of the Aleutian Islands.

**Fig 1:**
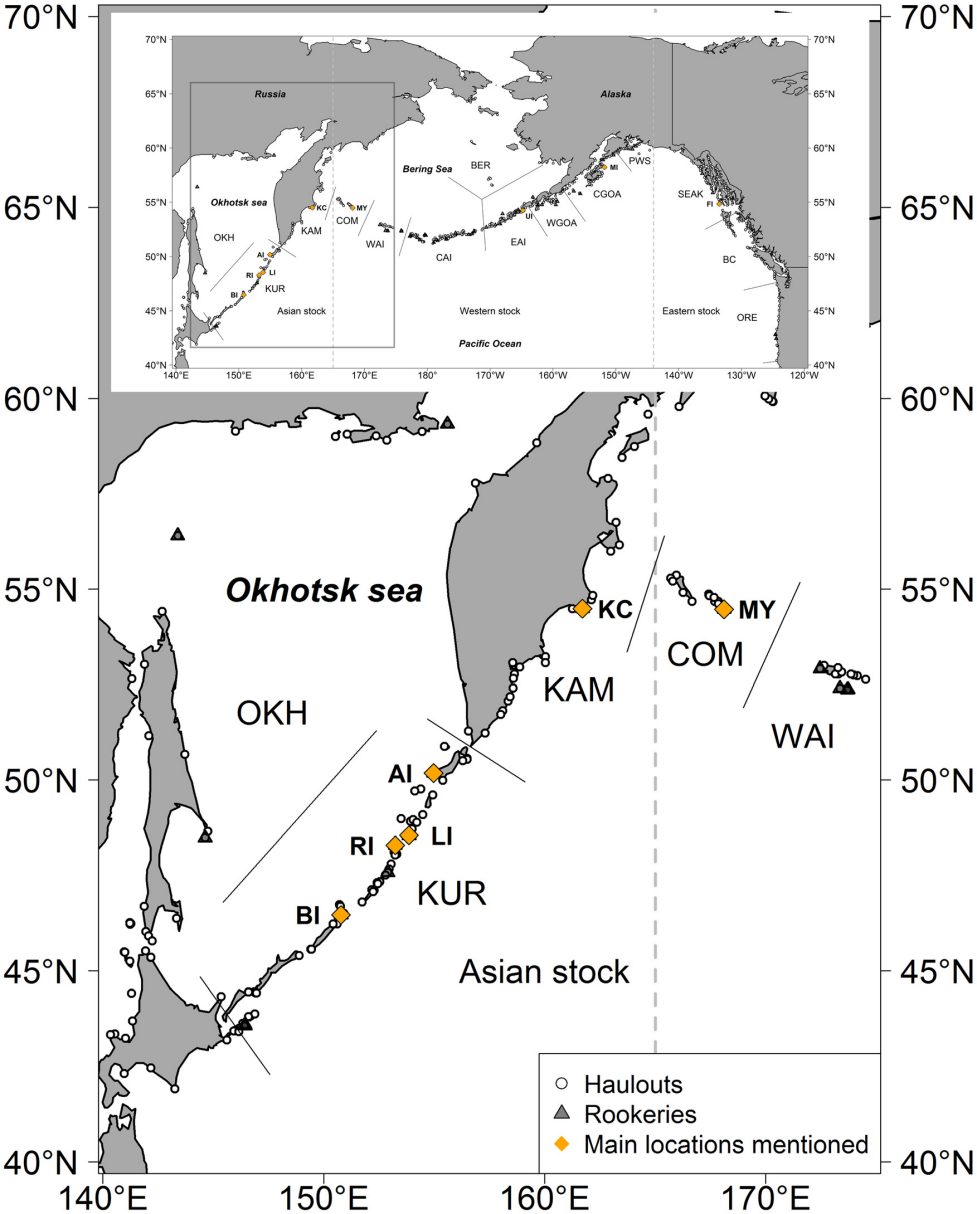
Steller sea lion terrestrial sites. Study sites: MY = Medny Island, KC = Kozlov Cape, AI = Antsiferov Island, LI = Lovushki Islands, RI = Raykoke Island, BI = Brat Chirpoev Island and other locations mentioned in this article: UI—Ugamak Island, MI—Marmot Island, FI—Forester Islands. Regional designations (after Baker et al. [5]): Sea of Okhotsk (OKH), Kuril Islands (KUR), Kamchatka Peninsula (KAM), Commander Islands (COM), Western Aleutian Islands (WAI), Central Aleutian Islands (CAI), Eastern Aleutian Islands (EAI), Bering Sea (BER), western Gulf of Alaska (WGOA), central Gulf of Alaska (CGOA), Prince William Sound (PWS), Southeastern Alaska (SEAK), British Columbia (BC), Oregon (ORE). Map generated using *maps* package [15] and the CIA World Data Bank II data (*world2HiresMapEnv*) available for R [16].

In far eastern Russia, there were approximately 16,000 Steller sea lions in 2005 [11], compared with 45,000 in the Alaskan portion of the western DPS and close to 50,000 in the eastern DPS at that time [12]. As in western Alaska, the overall population in Russia had declined dramatically between the 1970’s and the 1990’s. In Russian waters there are ten major Steller sea lion rookeries (i.e. sites where approximately 100 or more sea lions are born annually) and over 100 regularly used haulouts (Fig 1). Starting in the east, the Commander Islands archipelago consists of Medny, Bering and several small islands. The only currently active Steller sea lion rookery in the Commander Islands is situated on the southern tip of Medny Island. Five haulouts are spread out through the Commander Islands where there are also three northern fur seal rookeries. Medny Island is separated from Attu, the furthest west of the Aleutian Islands, by the 335 km wide Blizhny Strait. Between the Commander Islands and Kamchatka lies the 180 km wide Kamchatsky Strait. On the eastern coast of the Kamchatka Peninsula there is one Steller sea lion rookery, located approximately 2 miles offshore of Kozlov Cape. There are several Steller sea lion haulouts along the Eastern Kamchatka coast within a few hundred kilometers of the Kozlov Cape rookery. Five large Steller sea lion rookeries exist in the Kuril Islands, at Antsiferov Island, Lovushki Islands, Raykoke Island, Srednego Islands, and Brat Chirpoev Island. There are also about 40 haulouts spread throughout the Kuril Islands. At two rookeries, Lovushki and Srednego, Steller sea lions breed sympatrically with northern fur seals. In the Sea of Okhotsk there are three Steller sea lion rookeries.

The population trends at most of these Steller sea lion rookeries in Russia followed a similar pattern of decline between approximately 1975 and 1990, but during the 1990’s there was a divergence of trends across the region. In the Kuril Islands and in the Sea of Okhotsk the population started to increase and was still increasing in both areas through at least 2010 [13]. In Eastern Kamchatka the population was fairly stable with no discernible trend between 1990 and 2010, while on the Commander Islands the population has continued to decline through the present time [11, 14]. Starting in 1989, a program to brand Steller sea lion pups was begun in Russia, with a goal towards elucidating patterns of emigration. In the early 2000’s the project was expanded to include a regular program of branding and mark-resight observations at most of these rookeries.

The availability of consistently collected vital rates data from regions in Russia with different population trends over a ten year period provides a unique opportunity to investigate the relationship between population trends and survival rates. We focus here on the age-specific survival rates of Steller sea lions over the last decade at six rookeries and evaluate important differences in the factors that may affect Steller sea lion vital rates. The Commander Islands have been protected by a 30 mile no-fishing zone since the late 1950’s, and therefore the comparison of data from the Medny Island rookery in the Commander Islands with data from the Kuril Islands and Kamchatka may provide valuable insights for management of Steller sea lions, both in Russian and U.S. waters. Given the limited amount of information about Steller sea lions in the Western and Central Aleutian Islands, an understanding of the survival rates of Steller sea lions along the Russian coast may help researchers understand some of the proximal causes of continued decline in the Western Aleutian Islands.

## Materials and Methods

### Ethics statement

Animal handling and branding procedures were permitted by the Federal Supervisory Natural Resources Management Service (Rosprirodnadzor) of the Ministry of Natural Resources and Environment of the Russian Federation and approved by the Institutional Animal Care and Use Committee of the Alaska SeaLife Center. Permits allowing field research in protected areas were issued by Komandorsky and Kronotsky Biosphere State Nature Reserves (Commander Islands and Kozlov Cape), of the Ministry of Natural Resources and Environment of the Russian Federation, and Sakhalin and Kuril Regional Department of the Federal Agency for Fisheries of the Ministry of Agriculture of the Russian Federation (protected areas on and around the Kuril Islands).

### Marking

Data for this study were collected at six of the 10 major rookeries along the Russian coast: Antsiferov Island (AI), Lovushki Islands (LI), Raykoke Island (RI) and Brat Chirpoev Island (BI) in the Kuril Island chain; Kozlov Cape (KC) in Eastern Kamchatka, and Medny Island (MY) in the Commander Island archipelago (Fig 1).

Steller sea lions were marked when they were between two and five weeks of age with a unique alpha numeric ID using hot iron branding [17]. Cohorts from a single rookery were usually marked in one day. Between 1989 and 2001 pups were restrained by hand, but starting in 2002 the pups were anesthetized with isoflurane gas [18], which reduced sexing errors and resulted in improved brands that were easier to read when sea lions were resighted. Branding began in 1989 at three of the Kuril Islands rookeries: Lovushki, Raykoke, and Brat Chirpoev Islands (Fig 1, Table 1). Then after a gap of seven years, branding occurred in 1996 on all six of the rookeries: Medny Island, Kozlov Cape, Antsiferov, Lovushki, Raykoke and Brat Chirpoev Islands. Between 1996 and 2001 branding was performed annually at most of these rookeries. From 2002 through 2008 branding occurred every other year at each of the six rookeries (Table 1).

**Table 1:** Number of pups branded in each year on the six major Russian rookeries included in this work.

Year	Site	MY			KC			AI			LI			RI			BI			Total
	Sex	F	M	U	F	M	U	F	M	U	F	M	U	F	M	U	F	M	U	
1989	Original										97	103		74	65		108	81	11	539
	Corrected										102	98		84	55		115	76		530
1996	Original	46	54		25	25		42	58		57	43		48	52		56	44		550
	Corrected	46	54		26	24		46	54		56	44		48	52		54	46		550
1997	Original				20	30		54	46		51	49		40	60		47	53		450
	Corrected				20	30		54	46		50	50		41	59		50	50		450
1998	Original	49	38		28	22		44	50	6				49	51		44	56		437
	Corrected	50	37		27	23		46	48					48	52		46	54		431
1999	Original	55	45		26	24		22	28		45	55		48	52		58	42		500
	Corrected	54	46		26	24		22	28		47	53		48	52		58	42		500
2001	Original	56	39					50	50		45	55		42	57	1	32	44		471
	Corrected	57	38					51	49		44	56		45	55		32	44		471
2002	Original	46	39		22	28														135
	Corrected	45	40		22	28														135
2003	Original	26	28					39	61		37	40		48	52		36	64		431
	Corrected	25	29					40	60		37	40		48	52		36	64		431
2004	Original	55	45		26	24														150
	Corrected	55	45		26	24														150
2005	Original							38	62		51	51		49	51		45	55		402
	Corrected							39	61		53	49		49	51		46	54		402
2006	Original	44	56		20	30														150
	Corrected	44	56		21	29														150
2007	Original							46	54		52	48		41	59		53	47		400
	Corrected							46	54		52	48		41	59		53	47		400
2008	Original	53	47		26	24														150
	Corrected	54	46		27	23														150
Total original		430	391	0	193	207	0	335	409	6	435	444	0	439	499	1	479	486	11	4765
Total corrected		430	391	0	195	205	0	344	400	0	441	438	0	452	487	0	490	477	0	4750
Sex ratio original		0.52			0.48			0.45			0.49			0.47			0.50			0.49
Sex ratio corrected		0.52			0.49			0.46			0.50			0.48			0.51			0.50

Note: Original = based on sex determination during branding procedures. Corrected = correction after resights of animal at the age when sex may be unambiguously determined because of strong sexual dimorphism. MY = Medny Island, KC = Kozlov Cape, AI = Antsiferov Island, LI = Lovushki Islands, RI = Raykoke Island, BI = Brat Chirpoev Island. F = Females, M = Males, U = Sex was not determinated at branding time. Sex ratio calculated as the number of females divided by the total number of pups.

### Observation of marked sea lions

From 1989 to 2001 there were no regular efforts to resight the branded sea lions at five of the six rookeries. Observations during the breeding season were performed at Medny Island each year since 1996. Therefore, we excluded from our analysis observations made before 2002 on all sites except Medny Island. Beginning in 2002 we placed teams of at least 2 observers on each of five other major Steller sea lion rookeries. Observers were trained to identify brands and reproductive behavior and they monitored the rookeries for at least 2 months during the breeding season, from approximately the end of May/early June to mid-July/early August. Observations on rookeries were performed during daylight hours (starting at around 06:00 and continuing to 22:30, local time). Branded sea lions were filmed with still cameras or video to provide photo documentation of identifications. Additionally, all nearby haulout areas (Fig 1) in each geographic region were surveyed at least once per year during the breeding season (Table 2). In 2008 there was not proper resighting effort for Brat Chirpoev Island and Kozlov Cape, and we therefore omitted the year 2008 for these two sites in further analyses.

**Table 2:** Resighting effort (in days) at each rookery and its associated haulouts, each year, for the period 25 May—25 August.

Year	MY			KC			AI			LI			RI			BI		
	R	H		R	H		R	H		R	H		R	H		R	H	
	D	D	H#	D	D	H#	D	D	H#	D	D	H#	D	D	H#	D	D	H#
1997	71	0	0															
1998	73	0	0															
1999	78	1	1															
2000	49	11	4															
2001	73	0	0															
2002	80	9	6	52	9	6	39	14	10	38	15	11	39	15	11	38	5	4
2003	81	14	3	55	13	2	51	6	3	57	7	4	50	9	5	48	4	3
2004	69	39	2	52	34	1	56	0	0	55	0	0	53	0	0	51	0	0
2005	87	50	2	48	51	3	50	21	13	58	31	18	56	32	19	55	19	12
2006	80	48	2	77	48	2	58	4	2	58	4	2	56	4	2	58	0	0
2007	84	40	2	60	40	2	47	25	11	56	28	14	45	30	16	43	8	6
2008	76	12	10	0	0	0	45	13	11	41	14	12	24	15	13	0	0	0
2009	79	75	5	24	69	2	40	8	6	38	8	6	38	10	8	32	6	5
2010	75	26	8	71	23	6	56	6	6	54	6	6	54	6	6	50	2	2
2011	78	112	5	79	109	3	48	34	1	45	37	4	44	37	4	43	37	4

Note: natal rookeries: MY = Medny Island, KC = Kozlov Cape, AI = Antsiferov Island, LI = Lovushki Islands, RI = Raykoke Island, BI = Brat Chirpoev Island; H = all haulouts associated with particular rookery; D = effort represented as number of days (combined for the multiple haulout sites); H# = number of haulout sites surveyed.

### Data processing and analysis

We constrained our mark-resight analysis to animals born before 2009 and only included sightings in the summer period (May through August) of the years 2002 to 2011. Sightings made before 2002 (except Medny Island) were excluded because of the lack of regular sighting efforts before then. For the sea lions branded in 2001 and later, and resighted at least once by 2011, the large majority were resighted by the time they turned 4 years old (96.5%, SE = 1.06%). However, only 54.9% (SE = 7.87%) were resighted at age 1, and only 73.8% (SE = 4.38%) were resighted by the time they turned 2 years old. Approximately 87% (SE = 3.83%) of the resighted sea lions were seen by age 3. As a consequence, analyzing only first and second year data would likely to lead to underestimates of both survival and resight probabilities. To avoid this potential bias, we excluded branding cohorts with less than 3 years of post-branding observation history. Thus, even though we included observations through the year 2011, the last branded cohort we included was born in 2008. This resulted in 7341 resight records from 4765 marked animals.

Sighting records entered into the database were only used in our analysis if they were independently verified by at least three experienced observers. In the course of data verification we compared every photo confirmation to a master photo ID record. Master photo ID records were a compilation of photo confirmations for the same animal across all years that an individual was resighted and photographed since branding. This approach allowed us to substantially reduce misidentification errors. Steller sea lions are highly sexually dimorphic [19, 20] and sex was expected to be a significant factor for survival. We therefore separately assessed the level of sex identification errors during branding, because a high rate of sex identification errors could affect estimated vital rates. For the model selection we considered both datasets with and without sexing error correction. Additional details on this are included in the Supporting Information (S1 Text). In the final data set we included only animals with corrected sex data and excluded animals with unknown sex. This resulted in 7332 resights for 4750 marked sea lions.

We used a Cormack-Jolly-Seber model for open populations to estimate survival and sighting probabilities. The model estimates resighting probabilities (*p*) and survival probabilities (*Phi*) as unique functions of available covariates. We examined three main variables: Age, sex, and natal rookery (*site*). Model fitting was performed using MARK [21]. MARK software is a very powerful tool based on many theoretical probabilistic models for parameter estimates from marked animals when they are reencountered at a later time as dead recoveries, or live recaptures or resightings [22], and it has become a widely utilized solution for analyzing mark-recapture datasets. We also used the RMark package [23, 24] that provides a collection of functions for the R computation environment [16] that can be used as an interface to MARK. This approach simplified data input and model formulation.

Prior to modeling survival and resighting probabilities we estimated the overdispersion parameter (*c-hat*) for our most general model using the *median-c-hat* approach available in program MARK. For a general model we defined the survival probability as age*sex*site and the resight probability as age*sex*site + site*time. Overdispersion for our general model was estimated as 1.28 with 95% confidence intervals from 1.25 to 1.31. Even though the estimated overdispersion parameter was close to 1 and may be considered low [25] we used this inflation factor in our subsequent modeling

Survival is age dependent, but with up to 22 distinct age groups, we reduced the number of parameters by generating a B-spline matrix with four degrees of freedom for the prediction of age specific survival. The B-spline allows for the fitting of a highly flexible and well-constrained family of curves [26]. Four degrees of freedom were chosen because this enabled the model fit which minimized the AIC. We generated the basis spline using the *bs()* function in the R “splines” package [16]. Additional detail on the basis spline implementation can be found in the Supporting Information (S1 Text). An additional pup covariate was added to account for the unique role of first year survival.

We based model selection on testing all possible combinations of the main factors and their two and three way interactions. Additionally, for the resight probability (*p*) we examined the time effect with each year as a distinct covariate, and a time effect interaction with site was also considered. In order to examine the effect of variation in observation effort on differences in resight probabilities we also considered resight effort covariates such as effort on rookeries (RE), measured as the number of days spent on each natal rookery and haulout effort (HE) measured as the average number of observation days on each of the haulout sites associated with each natal rookery. Haulouts were considered to be associated with a rookery if any sea lions branded at that rookery had been observed on that particular haulout. We also included as a distinct covariate the number of surveyed haulouts (H#). We evaluated an interaction of number of days spent on haulouts with number of sites surveyed and the additive effect of the listed covariates. In order to narrow our question to a comparison of regions with similar population trends, we also performed the analysis replacing the site variable with a “region” (*region*) factor, in which the Kuril Islands group (KUR) consisted of the four Kuril Islands rookeries, the Eastern Kamchatka region consisted of just one rookery—Kozlov Cape, so it remained (KC), and the Commander Island region also consisted of just one rookery, Medny Island (MY). Along with the *region* covariate we averaged site-specific effort within the Kuril Islands group. Selection of models was based on comparing quasi AICc (QAIC with bias-correction for small sample size [25, 27]) weights.

Simultaneous exploration of all possible combinations of covariates considered for p and Phi would have been impossible to accomplish in any reasonable timeframe. One of the possible solutions to reduce computation time would be to first select a resight model [6, 10] and then using the best resight model perform selection of a survival model. This however, may potentially bias survival model selection towards the models that the resight probability selection procedure was based on. We tried to avoid this possibility by performing simultaneous evaluation of model candidates for resight and survival parameters. First we reduced the number of model candidates and selected the 20 best model candidates for *p* and the 20 best model candidates for *Phi*. To do this, we limited the Phi models to the most complex model (bs(Age)*sex*site) and the simplest null model (∼ *1*), and then selected the set of 20 best models for *p* according to QAICc. The same procedure was performed to select the 20 best *Phi* model candidates, using the most complex *p* model (bs(Age)*sex*site + time*site) and the simplest null model for *p* (∼ *1*). Using the selected set of *p* and *Phi* models we than evaluated combinations of models for both parameters simultaneously and then selected the best combination of models. The best combination of the two selected models was used to estimate resight and apparent survival probabilities across all sites.

## Results

### Cormack-Jolly-Seber models

We selected 20 of the 489 model candidates to describe resighting probability (Table 3) and 20 of the 159 model candidates to describe survival probability (Table 4). We used the selected models along with most simplest and most general model for *p* and *Phi* in our analysis and tested 484 combinations of models for both survival and resight parameters. The best 20 combinations of these models are provided in Table 5.

**Table 3:** Resight model candidate selection list.

Rank	Model	npar	QAICc	ΔQAICc	weight	QDeviance	chat
1	Ab:Ns:Sx + Pp:Ns + time:Ns	347	15823.84	0.00	0.86	3849.73	1.28
2	Ab:Ns:Sx + time:Ns	341	15827.58	3.74	0.13	3866.23	1.28
3	Ab:Ns:Sx + Pp:Ns + Sx:Ns + time:Ns	353	15832.66	8.82	0.01	3845.77	1.28
4	Ab:Ns + Sx:Ad + Pp:Ns + Sx:Ns + time:Ns	331	15841.38	17.54	0.00	3901.28	1.28
5	Ab:Ns + Pp:Ns + Sx:Ns + time:Ns	329	15851.15	27.31	0.00	3915.29	1.28
6	Ab:Ns:Sx + Pp:Ns + HE:H# + RE	293	15851.49	27.65	0.00	3991.77	1.28
7	Ab:Ns:Sx + Pp:Ns + HE:H#	292	15853.48	29.64	0.00	3995.86	1.28
8	Ab:Rg:Sx + Pp:Rg + time:Rg	294	15854.15	30.31	0.00	3992.32	1.28
9	Ab:Rg:Sx + Pp:Rg + Sx:Rg + time:Rg	297	15858.47	34.63	0.00	3990.31	1.28
10	Ab:Ns:Sx + Pp:Ns + Sx:Ns + HE:H# + RE	299	15860.45	36.61	0.00	3988.07	1.28
11	Ab:Ns:Sx + Pp:Ns + Sx:Ns + HE:H#	298	15862.59	38.75	0.00	3992.32	1.28
12	Ab:Rg:Sx + time:Rg	291	15866.54	42.70	0.00	4011.03	1.28
13	Ab:Ns + Sx:Ad + Pp:Ns + time:Ns	325	15868.23	44.39	0.00	3940.85	1.28
14	Ab:Rg + Sx:Ad + Pp:Rg + Sx:Rg + time:Rg	287	15871.56	47.73	0.00	4024.48	1.28
15	Ab:Ns:Sx + Pp:Ns + H#	292	15877.19	53.35	0.00	4019.57	1.28
16	Ab:Rg + Pp:Rg + Sx:Rg + time:Rg	285	15877.46	53.63	0.00	4034.59	1.28
17	Ab:Ns + Sx:Ad + Pp:Ns + Sx:Ns + HE:H# + RE	277	15878.12	54.28	0.00	4052.07	1.28
18	Ab:Ns + Sx:Ad + Pp:Ns + Sx:Ns + HE:H#	276	15880.27	56.43	0.00	4056.33	1.28
19	Ab:Ns:Sx + Pp:Ns + HE + RE	293	15880.28	56.44	0.00	4020.56	1.28
20	Ab:Ns + Pp:Ns + time:Ns	323	15882.21	58.37	0.00	3959.08	1.28

Note: Ab = basis spline on Age with df = 4, Sx = sex covariate, Pp = pup covariate, Ad = adult (1+) covariate (opposite to the pup covariate), Rg = region covariate, Ns = site (natal site), HE = effort on haulouts, H# = number of haulouts surveyed, RE = effort on the natal rookery, time = time (year) factor covariate. To allow models with multiple additive interaction terms we have removed the intercept.

**Table 4:** Survival model candidate selection list.

Rank	formula	npar	QAICc	ΔQAICc	weight	QDeviance	chat
1	Ab:Rg + Ab:Sx + Sx:Rg + Pp:Rg	301	15790.56	0.00	0.40	3913.96	1.28
2	Ab:Rg + Sx:Ad + Pp:Rg	297	15790.89	0.33	0.34	3922.73	1.28
3	Ab:Rg + Sx:Rg + Pp:Rg	298	15792.61	2.05	0.14	3922.34	1.28
4	Ab:Rg + Sx:Ad + Sx:Rg + Pp:Rg	300	15794.82	4.26	0.05	3920.33	1.28
5	Ab:Rg:Sx + Pp:Rg	307	15795.41	4.85	0.04	3906.13	1.28
6	Ab:Rg:Sx + Sx:Rg + Pp:Rg	310	15795.41	4.85	0.04	3899.80	1.28
7	Ab:Sx + Rg	290	15800.44	9.89	0.00	3947.04	1.28
8	Ab + Sx + Rg	287	15801.85	11.30	0.00	3954.77	1.28
9	Ab:Rg + Sx	293	15803.12	12.56	0.00	3943.39	1.28
10	Ab:Sx + Ns	293	15804.47	13.91	0.00	3944.75	1.28
11	Ab + Sx + Ns	290	15805.68	15.12	0.00	3952.28	1.28
12	Ab:Sx	288	15806.25	15.69	0.00	3957.06	1.28
13	Ab + Sx	285	15807.60	17.04	0.00	3964.72	1.28
14	Ab:Rg:Sx	304	15808.74	18.18	0.00	3925.80	1.28
15	age + Sx + Rg	304	15816.54	25.98	0.00	3933.60	1.28
16	Ab:Ns + Sx:Ad + Pp:Ns	312	15817.85	27.29	0.00	3918.01	1.28
17	age + Sx + Ns	307	15820.69	30.14	0.00	3931.42	1.28
18	age + Sx	302	15822.62	32.07	0.00	3943.91	1.28
19	Ab:Ns + Pp:Ns + Sx:Ns	316	15822.75	32.19	0.00	3914.44	1.28
20	Ab:Ns + Sx	305	15824.33	33.77	0.00	3939.28	1.28

Note: Ab = basis spline on Age with df = 4, Sx = sex covariate, Pp = pup covariate, Ad = adult (1+) covariate (opposite to the pup covariate), Rg = region covariate, Ns = site (natal site), time = time (year) factor covariate, age = age factor covariate. To allow models with multiple additive interaction terms we have removed the intercept.

**Table 5:** Selection list of survival and resight parameters model combinations.

Rank	Survival probability models	Resight probability models	npar	QAICc	ΔQAICc	weight	QDeviance	chat
1	Ab:Rg + Ab:Sx + Sx:Rg + Pp:Rg	Ab:Ns:Sx + Pp:Ns + time:Ns	133	15540.33	0.00	0.56	4013.05	1.28
2	Ab:Rg + Ab:Sx + Sx:Rg + Pp:Rg	Ab:Ns:Sx + time:Ns	127	15542.95	2.62	0.15	4027.95	1.28
3	Ab:Rg:Sx + Pp:Rg	Ab:Ns:Sx + Pp:Ns + time:Ns	139	15543.95	3.62	0.09	4004.37	1.28
4	Ab:Rg:Sx + Pp:Rg + Sx:Rg	Ab:Ns:Sx + Pp:Ns + time:Ns	142	15544.32	3.99	0.08	3998.59	1.28
5	Ab:Rg + Ab:Sx + Sx:Rg + Pp:Rg	Ab:Ns:Sx + Pp:Ns + Sx:Ns + time:Ns	139	15545.20	4.87	0.05	4005.62	1.28
6	Ab:Rg + Sx:Ad + Pp:Rg	Ab:Ns:Sx + Pp:Ns + time:Ns	129	15547.54	7.22	0.02	4028.45	1.28
7	Ab:Rg:Sx + Pp:Rg	Ab:Ns:Sx + time:Ns	133	15547.56	7.23	0.02	4020.28	1.28
8	Ab:Rg:Sx + Pp:Rg + Sx:Rg	Ab:Ns:Sx + time:Ns	136	15548.00	7.68	0.01	4014.57	1.28
9	Ab:Sx + Rg	Ab:Ns:Sx + Pp:Ns + time:Ns	122	15549.30	8.97	0.01	4044.52	1.28
10	Ab:Rg:Sx + Pp:Rg + Sx:Rg	Ab:Ns:Sx + Pp:Ns + Sx:Ns + time:Ns	148	15550.09	9.76	0.00	3992.04	1.28
11	Ab:Rg:Sx + Pp:Rg	Ab:Ns:Sx + Pp:Ns + Sx:Ns + time:Ns	145	15550.30	9.97	0.00	3998.41	1.28
12	Ab:Rg + Pp:Rg + Sx:Rg	Ab:Ns:Sx + Pp:Ns + time:Ns	130	15550.79	10.47	0.00	4029.65	1.28
13	Ab:Rg + Sx:Ad + Pp:Rg + Sx:Rg	Ab:Ns:Sx + Pp:Ns + time:Ns	132	15551.78	11.46	0.00	4026.55	1.28
14	Ab:Sx + Ns	Ab:Ns:Sx + Pp:Ns + time:Ns	125	15553.34	13.01	0.00	4042.43	1.28
15	Ab:Rg + Sx:Ad + Pp:Rg	Ab:Ns:Sx + Pp:Ns + Sx:Ns + time:Ns	135	15553.77	13.44	0.00	4022.39	1.28
16	Ab:Sx + Rg	Ab:Ns:Sx + time:Ns	116	15554.38	14.06	0.00	4061.86	1.28
17	Ab:Rg + Sx:Ad + Pp:Rg	Ab:Ns:Sx + time:Ns	123	15554.89	14.56	0.00	4048.07	1.28
18	Ab:Sx	Ab:Ns:Sx + Pp:Ns + time:Ns	120	15555.38	15.06	0.00	4054.69	1.28
19	Ab:Rg + Pp:Rg + Sx:Rg	Ab:Ns:Sx + Pp:Ns + Sx:Ns + time:Ns	136	15555.49	15.16	0.00	4022.06	1.28
20	Ab:Sx + Rg	Ab:Ns:Sx + Pp:Ns + Sx:Ns + time:Ns	128	15556.08	15.76	0.00	4039.04	1.28
461	age * Sx * Ns	age * Sx * Ns + time * Ns	515	16098.7	558.4	0.0	3761.4	1.28
462	1	age * Sx * Ns + time * Ns	280	16137.2	596.9	0.0	4304.9	1.28
483	age * Sx * Ns	1	237	16878.4	1338.1	0.0	5136.1	1.28
484	1	1	2	17345.0	1804.7	0.0	6082.9	1.28

Note: Ab = basis spline on Age with df = 4, Sx = sex covariate, Pp = pup covariate, Ad = adult (1+) covariate (opposite to the pup covariate), Rg = region covariate, Ns = site (natal site), HE = effort on haulouts, H# = number of haulouts surveyed, RE = effort on the natal rookery, time = time (year) factor covariate. To allow models with multiple additive interaction terms we have removed the intercept.

The pooling of all Kuril Islands rookeries into one regional covariate did not improve the resight model, and models with a regional covariate had a higher QAICc (see Table 3). This suggested that resighting probabilities varied significantly among each of the Kuril Island rookeries. Models with any of the effort covariates instead of time or site also had higher QAICc suggesting low impact of those covariates on resight rates (see also Supporting Information S1 Text). However, resight models with effort covariates as well as models with a regional covariate were considered as a candidates for final testing (see Table 3). Using the region instead of site covariate, however, improved the survival model. All of the top survival model candidates included the region rather than site covariate (see Table 4).

The best resight model suggested age and sex dependent resight rates for adults, but the sex difference in resight probability for pups was negligible. The best survival model suggested that age specific survival probability was unique for each region but differences in survival between sexes was mostly uniform across all rookeries, and also depended on age (model 1 Table 5).

Additional details on the resight rate description and model selection are provided in the Supporting Information (S1 Text).

### Resighting probabilities

**Fig 2:**
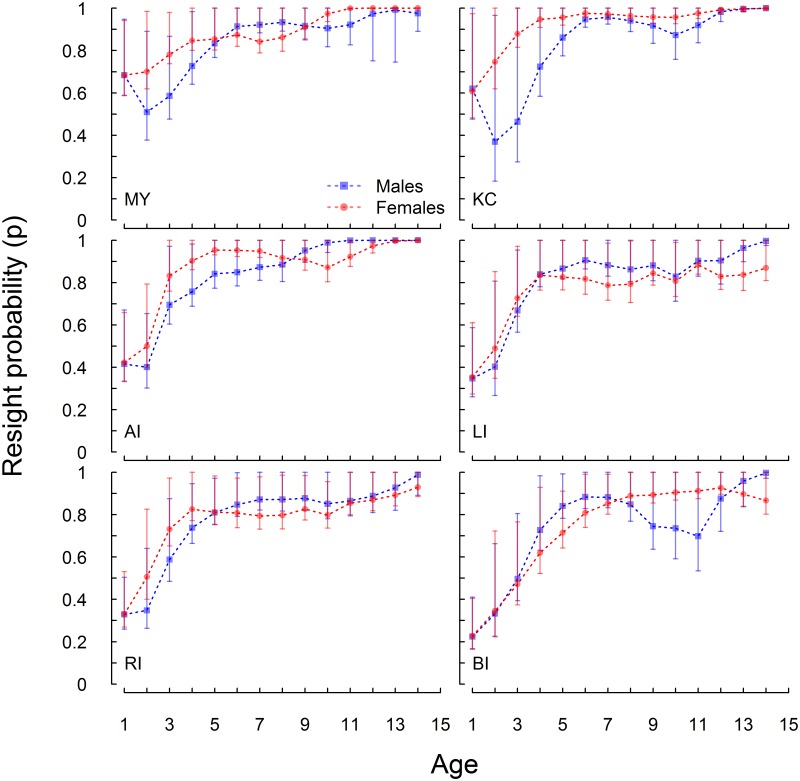
Average age specific resight probabilities of females (red circles) and males (blue squares). Error bars = 95% confidence intervals. Medny Island (MY), Kozlov Cape (KC), Antisferov Island (AI), Lovushki Islands (LI), Raykoke Island (RI), Brat Chirpoev Island (BI).

Although age specific resight patterns differed between all sites, resight probability generally increased with age, with the exception that on Medny Island and Kozlov Cape the male resight probability for pups was higher than in the second year (Fig 2). Resighting rates were higher on Medny Island and Kozlov Cape than on all Kuril Islands rookeries.

### Apparent survival rates

**Fig 3:**
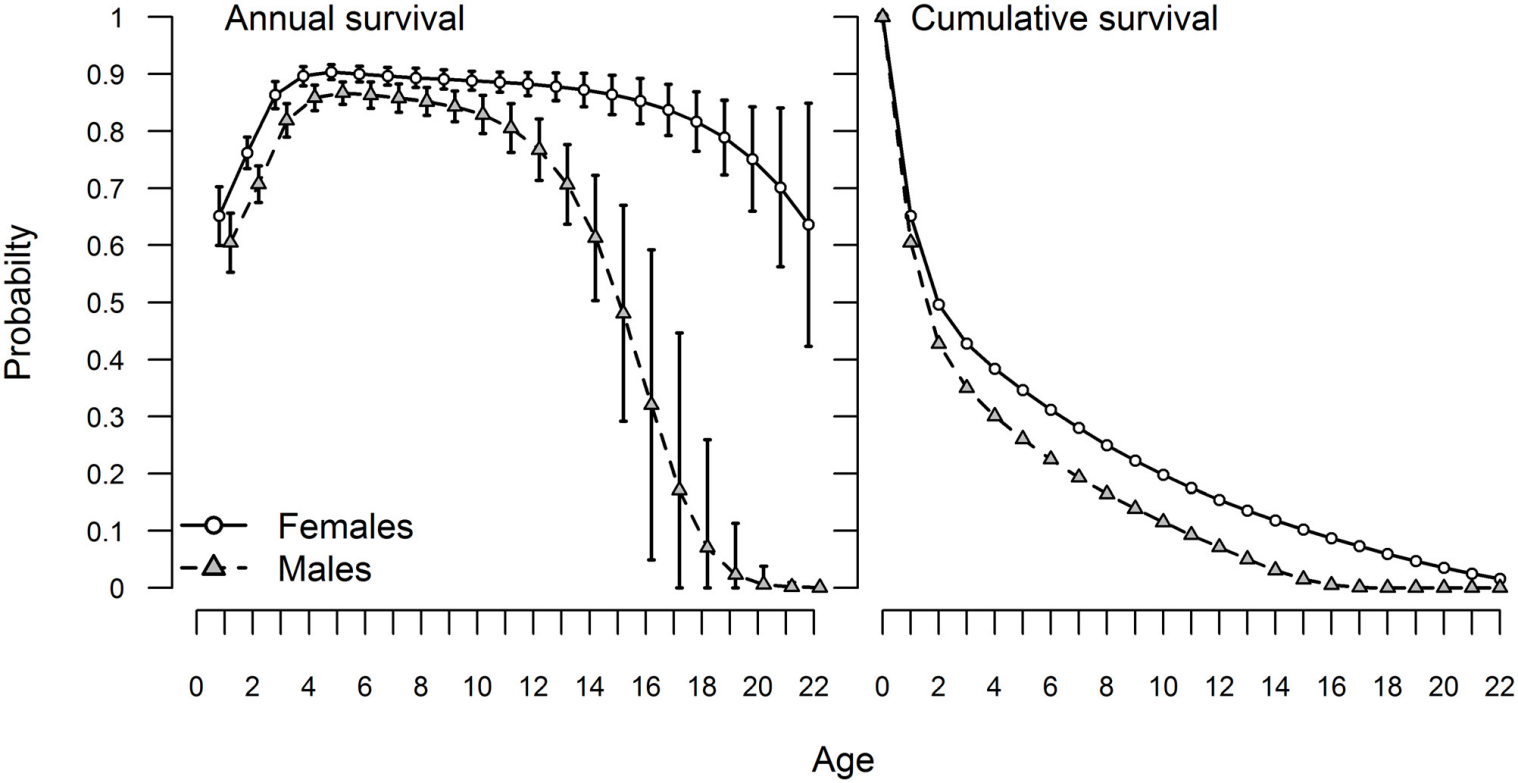
Annual and cumulative age and sex specific survival for the Kuril Islands, through 22 years of age (error bars = 2 SE). Note that the survival estimates are plotted at the maximum age of each age class, e.g. the survival of age 0 sea lions to age 1 is plotted at 1.

On the Kuril Islands rookeries, the first cohort was branded in 1989, enabling us to construct age specific survival up to age 22 (Fig 3, S1 Table), nearly the maximum lifespan for Steller sea lions. Therefore, we used the Kuril Islands data to demonstrate the general pattern of age-specific survival in this growing population area. Annual survival (both sexes) increased from pup birth to age 4 (Fig 3), then reached a plateau. Maximum annual survival occurred between the age groups of 3–4 to 7–8. After age 8–9 annual survival decreased slowly for females until the 14–15 yr. old age group, and then survival more rapidly decreased, especially after age 17–18. Annual male survival rapidly went from 0.77 (SE = 0.03) for the 11–12 age group to virtually zero for the 21–22 age group. Cumulative survival for males reached nearly zero at the 17–18 age group, while female survival was still around 0.06 at that age (Fig 3, S2 Table). By age 22, cumulative survival for males was practically zero, while female cumulative survival was still around 0.015.

**Fig 4:**
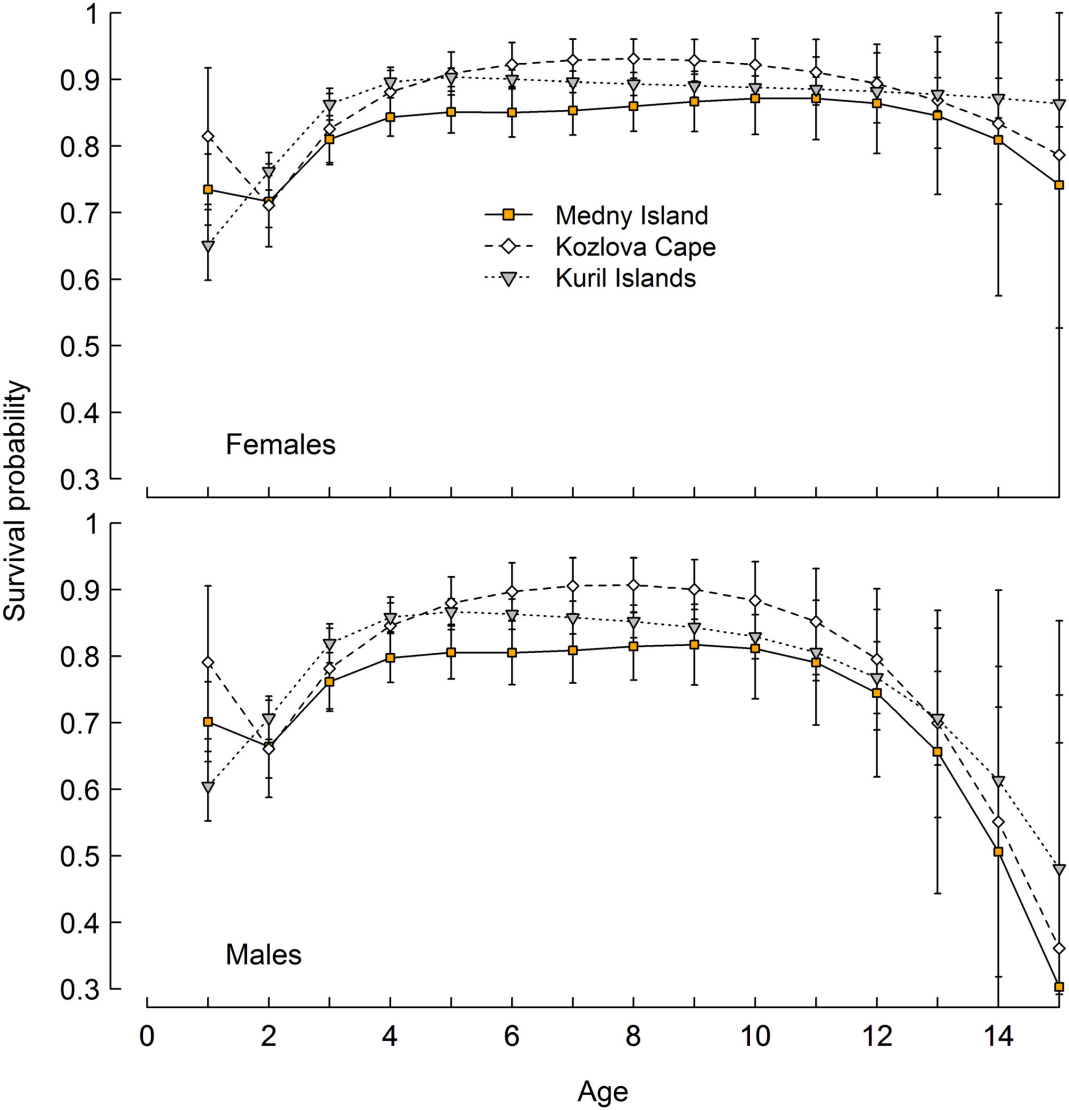
Age specific annual survival for females and males until age 15 in the Commander Islands, Eastern Kamchatka and Kuril Islands (error bars = 2 SE). Note that the survival estimates are plotted at the maximum age of each age class, e.g. the survival of age 0 sea lions to age 1 is plotted at 1.

The same general pattern was found in all three regions we studied. Estimated survival probabilities (until the 14–15 age group) between all regions confirmed higher annual survival rates in females (0.65–0.93) than males (0.30–0.91). Annual survival was highest for the 3–4 through 13–14 year old age groups of females (0.81–0.93) and 3–4 to 10–11 year old age groups of males (0.74–0.91) across all regions (Fig 4).

Steller sea lions on Medny Island and Kozlov Cape displayed a dip in survival in second year relative to first and third year survival. On Medny Island, female first year survival (0.73, SE = 0.3) was slightly higher than second year survival (0.72, SE = 0.02), while third year survival (0.81, SE = 0.02) was much higher than first and second year survival. Similarly, male survival on Medny Island over the first year (0.70, SE = 0.03) was higher, but not significantly, than the second (0.66, 0.22), while third year survival (0.76, SE = 0.02) was higher than first and second year survival. Survival rate for females on Medny Island did not change significantly between four and 13 years of age, and was likely stable past age 13, but the higher estimation error for the 13–14 and 14–15 age groups prevented comparison. For males at Medny Island, this period of stable high survival was somewhat narrower and only spanned from the 3–4 through the 10–11 year old age groups. On Kozlov Cape, female first year survival (0.81, SE = 0.05) was higher than second year survival (0.71, SE = 0.03), and similar to third year survival (0.83, SE = 0.03). Likewise, first year survival for males (0.79, SE = 0.06) was higher than second year (0.66, SE = 0.04) on Kozlov Cape (Fig 4). The annual survival rate for both males and females at Kozlov Cape did not change greatly from ages 4 to 10.

In contrast to Medny and Kozlov Cape, first year survival for females (0.65, SE = 0.03) and males (0.60, SE = 0.03) was lower than second year survival (0.76 SE = 0.01 and 0.71 SE = 0.01) in the Kuril Islands. The female survival rate increased steadily until age 3–4. On the Kuril Islands female survival plateaued at this age and did not change greatly (0.86–0.91) until the 14–15 age group (Fig 4). Male survival followed the same pattern as female survival, but with a shorter period of relative stability and high survival (0.81–0.87) for the 3–4 to 10–11 age groups (Fig 4).

**Fig 5:**
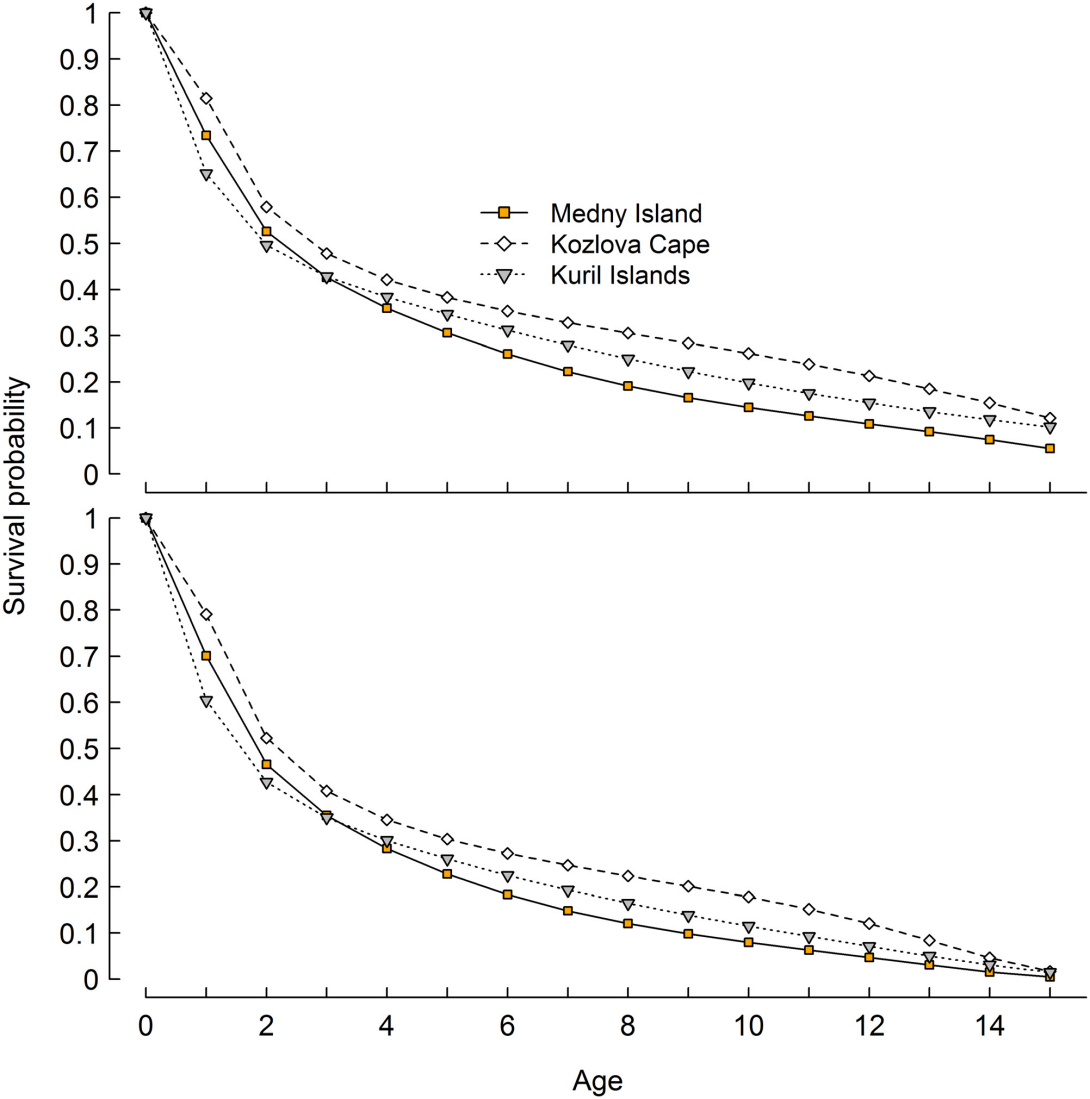
Age specific cumulative survival for females and males until age 15 in the Commander Islands, Eastern Kamchatka and Kuril Islands.

Pup survival for both sexes (0–1 age group, Fig 4) was different between all regions. However pup survival on Medny Island (0.70–0.73) and Kozlov Cape (0.79–0.81) was higher than on the Kuril Islands (0.60–0.65). However, female juvenile survival between 1–2 years of age was higher in the Kuril Islands (0.76, SE = 0.01) than on Medny Island (0.72, SE = 0.02) and Kozlov Cape (0.71, SE = 0.03). Female survival from age 2 to 3 was also significantly higher in the Kuril Islands (0.86, SE = 0.01), than on Medny Island (0.81, SE = 0.02) and Kozlov Cape (0.83, SE = 0.03). Survival from age 3 to 4 and 4 to 5 was about the same between the Kuril Islands (0.90–0.90, SE = 0.01) and Kozlov Cape (0.88(SE = 0.02)–0.91(SE = 0.02)) and higher than on Medny Island (0.84(SE = 0.01)–0.85(SE = 0.02)). For the 5–6 through 8–9 year old age groups, survival estimates were higher on Kozlov Cape (0.92–0.93) than in the Kuril Islands (0.89–0.90) and lowest on Medny Island (0.85–0.87) (Fig 4). Cumulative survivorship of females on Medny Island became lower than in other regions after age 4 (Fig 5). Cumulative survival of females to age 14 was twice as low on Medny Island than in the Kuril Islands and Kozlov Cape (Fig 5).

Survival of males followed the same pattern as for females and it was higher for age groups 1–2 and 2–3 in the Kuril Islands than on Medny Island and Kozlov Cape, and then became higher on Kozlov Cape (0.88–0.91) than in the Kuril Islands (0.83–0.86) and Medny Island (0.80–0.82) for the ages 5–6 to 9–10. After age 12, error estimates were too large and obscured differences between regions (Fig 4).

## Discussion

### Pattern of age/sex specific survival

Based on the Kuril Islands data, where earlier cohorts were marked and observed, it was possible to use the estimated survival rates to obtain total survivorship curves up to 22 years of age. Female survival was higher for all ages, as is typical for mammals with high levels of sexual dimorphism [28, 29]. Annual survival increased with age, reaching a plateau by four years of age, as seen with Steller sea lions elsewhere [6]. Sex-specific survival curves greatly diverged around ages 10–12. This is consistent with reproductive behavior of Steller sea lions and corresponds to the age at which Steller sea lion males begin to participate actively in reproduction on major rookeries [30–32]. The males establish territories by age 8–10 and hold them on average for 4–8 years [30, 33]. Sex differences in survival are usually attributed to male-male competition, higher nutritional requirements linked to larger body size and to mating strategies [34, 35]. Survival may be affected in several ways: direct injures caused by male-male aggression [36] and the cumulative effect of physical deterioration related to fasting, vigilance and breeding, reducing survival ability in late fall. According to our estimates, male survivorship by age 17 is virtually zero, suggesting that most males attempt to hold territories until the end of their lives. Annual survival of females, in contrast, is not affected greatly by reproduction, which begins between the ages of 3–6, and survival remains high from four until 18–20 years of age (Fig 3).

Previous studies on Steller sea lion vital rates highlighted differences in survival between sexes, and discussed reasons that may explain observed differences [6, 37]; however, those studies did not include the older ages when males are holding territories. Differences between sexes in annual survival ranged from 0.02–0.06 until the age 10 in our study, which is very close to the results from previous Steller sea lion studies [6, 37]. However, differences in annual survival between sexes in our study for the older ages clearly showed that survival of reproductively active males rapidly decreased after age 10. For the ages from 10 to 22 the difference in annual survival between sexes ranged from 0.08–0.77. A decrease in age–specific male survival soon after beginning reproduction was also found in northern elephant seals [38], a species with a similarly challenging polygynous mating system. Steller sea lion males had a relatively narrow window of a few years after successfully establishing a territory for the successful production of offspring. In contrast, many mammalian females can control the extent to which they invest energy in reproduction versus survival [39, 40]. This trade-off may also occur in Steller sea lion females, as there has been a wide range of variation in natality across the Steller sea lion range [8, 41].

### First and second year survival across regions

We found regional variation among the Russian rookeries in the overall survival patterns described above. Pup survival through age one at Medny Island (Commander Islands) and Kozlov Cape (Eastern Kamchatka) (0.70–0.82) was higher than on the Kuril Islands (0.60–0.65; Fig 4) and close to the level of pup survival estimated for the Gulf of Alaska Steller sea lion population in the pre-decline period (around 0.80) as estimated by Holmes et al. [8]. This level is also similar to the estimated pup survival on Marmot Island (0.73) during the decline [37]. First year survival on Medny Island and Kozlov Cape is also higher than in other stable and recovering regions such as the Eastern Aleutian Islands (0.52–0.57) [6]. Interestingly, unlike the Kuril Islands and most other places where age specific estimates have been obtained, second year survival decreased on Medny Island and Kozlov Cape. The same pattern was observed on Marmot Island during the population decline [37], and recently was found in the Western [10] and Eastern [42] Gulf of Alaska.

High pup survival on Medny Island and Kozlov Cape was an unexpected result, in light of the current negative population trends there [11]. Fritz et al. [10] suggested that high pup survival could be the result of longer average periods of maternal care, lower predation rates, or a combination of these and other factors. First year survival for many animals depends on early conditions, for example on body mass at birth or within the first weeks of growth [6, 43], which in turn is often closely related to adult female condition. Recent studies of maternal attendance patterns indicate that over the period of our study, Medny Island females had a longer postpartum period, the time after pup birth until the first foraging trip, than at other Steller sea lion rookeries, suggesting that adult females there were in better condition and capable of fasting and suckling their pups for a greater length of time during the first suckling bout [13].

One explanation for the unexpected result of high pup survival in areas with flat or negative population trends may be found in the behavior of Steller sea lion females. Most adult females appear on the rookery without their offspring from the previous year. In some cases, however, females do nurse their pups beyond one year. Pitcher and Calkins [32] reported that on Marmot Island 28% of adult females were accompanied by juvenile offspring, and in some cases this relationship extended up to 3 years. Extreme cases were documented by Mamaev et al. [44] on Medny Island, where a cow nursed a 4 year old female offspring, which in the same summer was nursing her own newborn pup. Preliminary results from Medny Island indicated that about 33% of females there did not give birth every year, often skipping a year and sometimes even two years between births, and appeared on the rookery or haulouts with offspring from the previous year [45]. The average age of weaning for Steller sea lions is unknown, though it has been suggested to be around one year [32, 46–48]. One might expect that when an adult female extends the nursing period beyond one year that this results in increased second year survival for her offspring, as it was highlighted in other Steller sea lion studies [6, 42]. In fact, we observed the opposite: Medny Island and Kozlov Cape, rookeries with the most delayed weaning according to our preliminary results [45], were characterized by the lowest second year survival rates. Perhaps observations of extended nursing periods are a consequence of lower reproductive rates on Medny Island [49], with many females skipping reproduction for at least one year, lessening the need to abruptly wean their offspring at the end of its first year. It is possible that weaning occurs shortly after the summer, and therefore the period of extended weaning is not very long and has little positive impact on winter survival of the offspring. In contrast, preliminary results for reproductive rates on at least one of the Kuril Islands appear higher than on Medny Island [49]. Females that breed in consecutive years have less time to recover their body condition, and less resources to invest in their next pup, consequently first year survival of those pups might be lower. If a greater fraction of reproductive age females skip birth years on Medny Island, this could partly explain their higher apparent body condition as indicated by longer post-partum periods [13].

### Juvenile survival across regions

**Fig 6:**
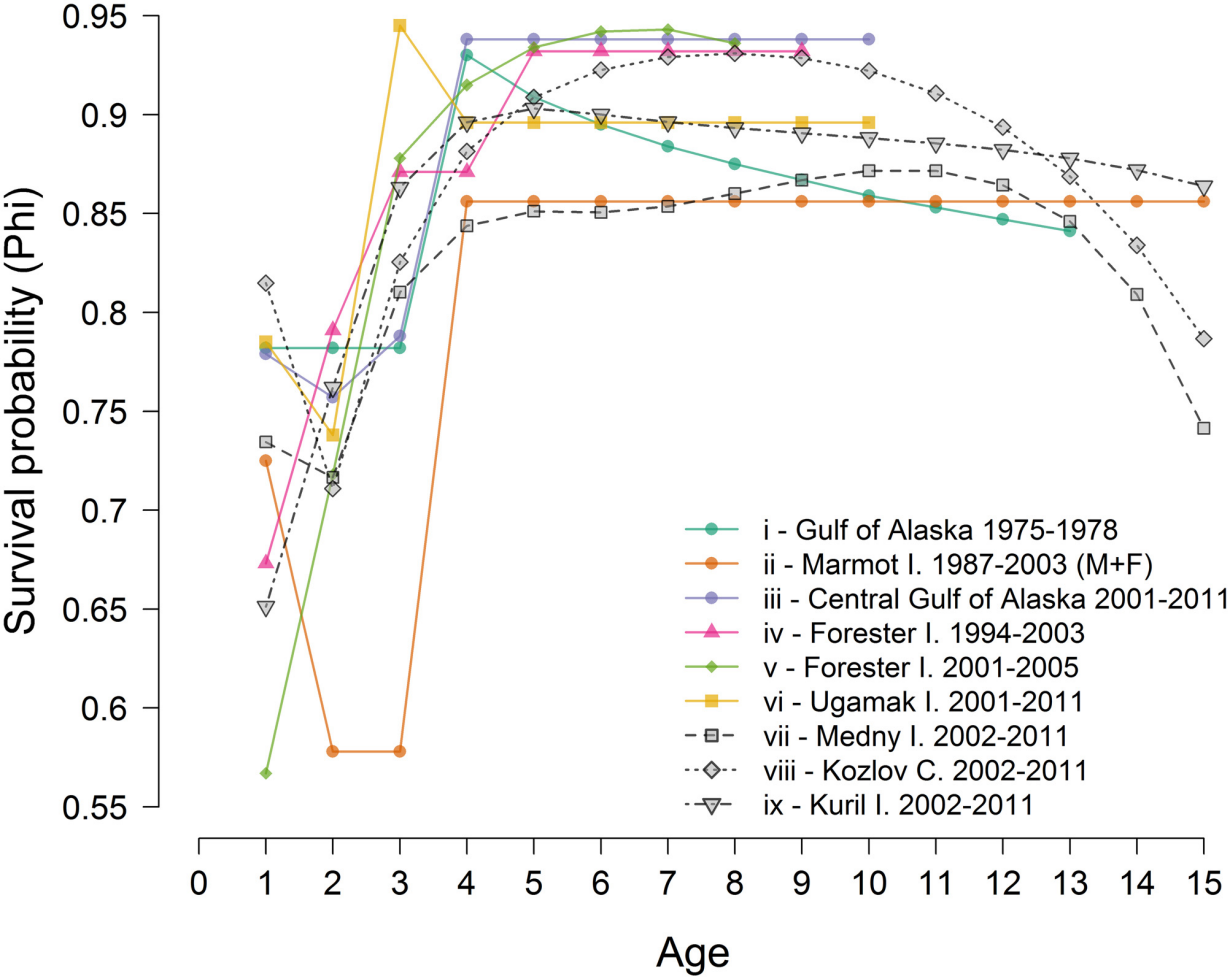
Apparent annual survival in different time periods and geographic regions. (i) pre-decline in central Gulf of Alaska [54]; (ii) pooled male and female survival during decline in Marmot I. [37]; (iii) Central Gulf of Alaska females post-decline [10]; (iv) Eastern Stock females survival 1994—2003 [37] and (v) more recently [6]; (vi) Eastern Aleutian females 2001—2011 [10]; (vii, viii, ix) female survival at Russian rookeries (this study). Note that the survival estimates are plotted at the maximum age of each age class, e.g. the survival of age 0 sea lions to age 1 is plotted at 1.

**Fig 7:**
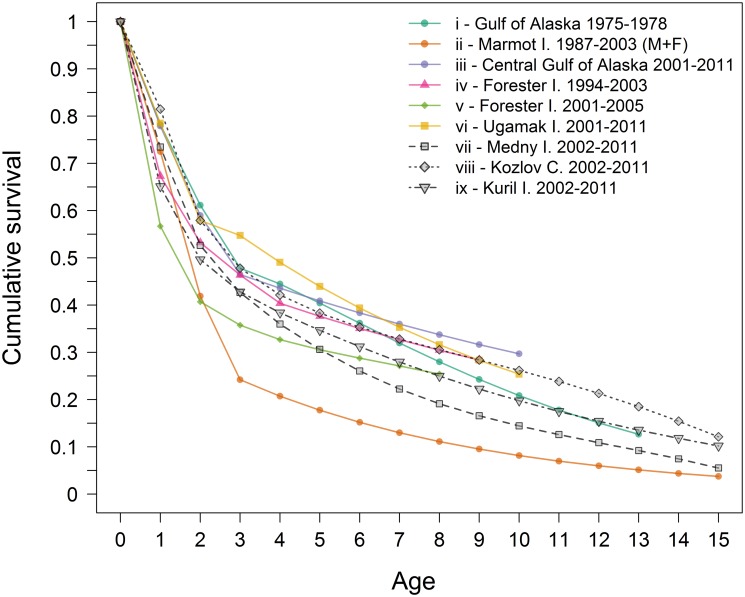
Cumulative survivorship in different time periods and geographic regions. (i) pre-decline in central Gulf of Alaska [54]; (ii) pooled male and female survival during decline in Marmot I. [37]; (iii) Central Gulf of Alaska females post-decline [10]; (iv) Eastern Stock females survival 1994—2003 [37] and (v) more recently [6]; (vi) Eastern Aleutian females 2001—2011 [10]; (vii, viii, ix) female survival at Russian rookeries (this study).

Juvenile survival is the vital rate likely most sensitive to persistent changes in environmental conditions [50]. It is accepted that the Steller sea lion decline in the late 1970s and 1980s was primarily due to low survival of juveniles [8, 51–54]. Our estimates for juvenile survival in all regions in this study (Fig 6 and Fig 7) are similar to estimates for the pre-decline [54] and recent post-decline [8] periods in the Central Gulf of Alaska (CGOA) as well as for Eastern Aleutian Islands [10] and also similar (Fig 7) to recent estimates for Southeast Alaska [6]. They are higher than those in the CGOA during the period of decline [8, 37]. Regional differences in the Russian population are significant, indicating that juvenile survival is higher for the recovering Kuril Islands than in Kozlov Cape and Medny Island (Fig 4), but the differences are smaller than those reported in Alaska between decline and pre- and post-decline periods.

The reasons for the regional variation we observed in juvenile survival are unclear. The Commander Islands region (Medny Island rookery) are protected by a 30 mile no-fishing zone, while Kozlov Cape in Eastern Kamchatka is located in an area with intensive commercial fisheries, but survival in both areas was similar. This suggests that other factors may be important, for example seasonal food availability around the islands, level of predation, winter weather conditions, etc. (see for example [55–57]). Our unpublished survey data from recent years shows that very few Steller sea lions remain in the Commander Islands over winter, and the nearest occupied haulouts are found along the Eastern Kamchatka coastline. Therefore, juveniles born on Medny Island may spend considerable time in the same areas as those born on Kozlov Cape, which could explain their similar survival rates.

### Adult survival across regions

Average pooled (female and male) adult survival (> 3 years old) on Medny Island (0.78) was less than estimated for Marmot Island (avg. 0.86) in the Gulf of Alaska during the decline. Typically in long-lived mammals, adult survival is the vital rate that shows the slowest response to long-term impacts [8, 50, 58], and changes in survival rates are likely more important in driving Steller sea lion population dynamics than birth rates. Low adult survival on Medny Island may be due to some long-term negative effect on adult survival that is potentially preventing the Medny population from recovering, but estimates of birth rates are required before making a proper conclusion. Lower adult survival may suggest that these sea lions experience predation, disease or food limitation during the winter time, as there is no evidence of nutritional stress during summer [13, 59]. We have not observed any overt sign of unusual disease mortality [36], and predation seems an unlikely explanation given that killer whales have never been observed to prey upon Steller sea lions during summer on Medny Island, despite frequent observations of predation upon juvenile male northern fur seals there. The fur seals dramatically out-number Steller sea lions on Medny Island, even in winter when most fur seals have migrated south [11, 60]. Adult survival may have been influenced by other factors such as fisheries interactions, for example entanglement in nets [61], and although data on this are lacking studies are underway to examine this possible threat.

Survival rates at all adult ages were lower on Medny Island than on the other rookeries (Fig 4). Adult survival rates on the Kuril Islands were higher than on Medny Island, but, surprisingly, lower than on Kozlov Cape. The fact that the Kuril Islands, with the most positive population trends, did not have the highest pup or adult survival is evidence for the hypothesis that variation in birth rates might be a key factor in the divergent population trends in far eastern Russia. Specifically, on Medny Island, low female survival after age 4, together with a strategy of pupping only once in two or even three years is likely limiting pup production. Explaining the surprisingly high survival rates of pups and adults on Kozlov Cape is difficult, as resighting efforts were more infrequent on this rookery.

### Conclusion

Survival rates of Steller sea lions differed between the Kuril Islands, Kamchatka and Commander Islands regions, though not necessarily as predicted by population trends. This leads us to suggest that the divergent population trends are influenced by differences in birth rates. In particular, recent high pup survival on the Commander Islands and Kamchatka Coast is likely a consequence of less frequent (e.g. biennial) reproduction. Juvenile survival is lower in the Commander Islands and Kamchatka than on the Kuril Islands, which agrees with the other data that suggest that, at least in early years, Commander Islands and Kamchatka juveniles share feeding regions and experience similar environmental conditions. All age-specific survival rates, except pup survival, were lower in the Commander Islands than in all other regions, and may be the main reason for the lack of recovery there. However, in order to tease apart the proximal causes for variation in population trends, it is important to estimate both birth and migration rates, and incorporate those into a comprehensive demographic model. Therefore, the next steps in our research are to identify those parameters which explain the most variability in the population structure and abundance and to test hypotheses for the ultimate causes of population decline or recovery for the Russian population of Steller sea lions.

## Supporting Information

S1 Text Methods and Model Selection. (PDF)

S1 Fig Level of brand identification errors, with 95% confidence intervals for the binomial distribution with Agresti-Coull approximation. (TIFF)

S2 Fig Level of sexing errors, with 95% confidence intervals for the binomial distribution with Agresti-Coull approximation. (TIFF)

S3 Fig Effect of sex error correction on survival rate estimation for females. Estimation for Medny Island (MY), Kozlov Cape (KC), Antisferov Island (AI), Lovushki Islands (LI), Raykoke Island (RI), and Brat Chirpoev Island (BI).
(TIFF)

S4 Fig Effect of sex error correction on survival rate estimation for males. Estimation for Medny Island (MY), Kozlov Cape (KC), Antisferov Island (AI), Lovushki Islands (LI), Raykoke Island (RI), and Brat Chirpoev Island (BI).
(TIFF)

S5 Fig Effect of including cohorts with less than 4 years of resight history on survival rate estimation for females. Estimation for Medny Island (MY), Kozlov Cape (KC), Antisferov Island (AI), Lovushki Islands (LI), Raykoke Island (RI), and Brat Chirpoev Island (BI).
(TIFF)

S6 Fig Effect of including cohorts with less than 4 years of resight history on survival rate estimation of males. Estimation for Medny Island (MY), Kozlov Cape (KC), Antisferov Island (AI), Lovushki Islands (LI), Raykoke Island (RI), and Brat Chirpoev Island (BI).
(TIFF)

S7 Fig Seasonal cumulative resight probability (boxes and black lines) and probability that observation occurred during a specific week of the year (blue lines). Medny Island (MY), Kozlov Cape (KC), Antisferov Island (AI), Lovushki Islands (LI), Raykoke Island (RI), Brat Chirpoev Island (BI).
(TIFF)

S8 Fig Time specific resight probability for females. Medny Island (MY), Kozlov Cape (KC), Antisferov Island (AI), Lovushki Islands (LI), Raykoke Island (RI), Brat Chirpoev Island (BI).
(TIFF)

S9 Fig Proportion of resightings that were made on haulouts. Medny Island (MY), Kozlov Cape (KC), Antisferov Island (AI), Lovushki Islands (LI), Raykoke Island (RI), Brat Chirpoev Island (BI).
(TIFF)

S10 Fig Flexibility of basis spline curves with different degrees of freedom in females. Blue dotted line in each cell represents the best survival model with df = 4. Medny Island (MY), Kozlov Cape (KC), all Kuril Islands (KUR).
(TIFF)

S11 Fig Flexibility of basis spline curves with different degrees of freedom in males. Blue dotted line in each cell represents the best survival model with df = 4. Medny Island (MY), Kozlov Cape (KC), all Kuril Islands (KUR).
(TIFF)

S1 Table Annual age and sex specific survival and standard error estimations of Steller sea lions branded along the Russian coast during the period 1989–2008, and resighted in the period 1997–2011 (Medny Island) and 2002—2011 (all other rookeries). Estimates based on model 1 in Table 5.
(PDF)

S2 Table Cumulative age and sex specific survival with 95% confidence intervals of Steller sea lions branded along the Russian coast during the period 1989–2008, and resighted in the period 1997–2011 (Medny Island) and 2002—2011 (all other rookeries). Estimates based on model 1 in Table 5.
(PDF)
