# Supplementary material for: Age Specific Survival Rates of Steller Sea Lions at Rookeries with Divergent Population Trends in the Russian Far East
Source: PLoS One. 2015 May 27;10(5):e0127292. doi: 10.1371/journal.pone.0127292 (PMC4446299; doi:10.1371/journal.pone.0127292)
Supplement: S1 Text — (PDF) [file pone.0127292.s001.pdf]

S1 Text: Methods and Model Selection

Data verification, correction and data selection

Over the course of over 20 years of branding, albeit with significant gaps in the early years, and 10 years of dedicated re-sighting efforts, we have collected valuable demographic data on over 5000 animals spanning nearly a full life-span of a Steller sea lion. This effort has involved dozens of field workers both for marking and observers for resighting. Consequently, the field methodology has developed over the years, and in analyzing the data it was important to take into consideration the effect of an evolving and generally improving methodology. The rates of sex identification error during branding and brand misidentification on resightings decreased significantly with time. The highest level of resighting error occurred in 2002, in part because it was the first year of dedicated resighting effort for mark-resight and the majority of observers were not very familiar with brands. Also, some brands were difficult to read because pups branded previous to 2002 were not anesthetized. A second important factor in the decrease in identification errors was an improved data entry process. In 2005, the data entry procedure was integrated into a specially developed database in MS Access. Probably the most critical improvement was the incorporation of a master brand ID catalogue photo viewer for use in the field, which allowed for the collection of animal information by observers on rookeries with the ability to compare field photos with master photo ID images. The level of brand identification error was significantly higher for the period prior to 2006 (Chi-squared = 192, df = 1, p-value < 0.05). The highest level of misidentification occurred in the earliest years of the study 2002–2003 (see S1 Fig.). Only records that could be corrected were used in our analyses. All other observations with no photo or video confirmation were not used. We separately assessed the level of sex identification errors during branding, because a high rate of sex identification errors (see S2 Fig.) could affect estimated vital rates. For the model selection we considered both datasets with and without sexing errors correction, and then evaluated the effect of sex identification corrections on the vital rates estimates. Cohorts born before 2002 had a significantly higher rate of sex misidentification at time of branding (S2 Fig.) (Chi-squared = 35.7, df = 1, p-value < 0.05). A total of 1,872 branded animals were resighted at least once at an age when we could determine their sex from unambiguous secondary sexual characteristics. Of those that had been classified as males during branding (N = 890), 5.5% were later identified as females. Among those recorded as females during branding (N = 982), 2.4% were later identified to be males. As with the brand misidentification, sex misidentification was highest during the earliest years of the study: among surviving animals branded before 2001 (N = 618), 13.8% of those classified as males at branding turned out to be females (N = 247), and 6.5 % (N = 371) of those classified as females at branding turned out to be males. We consider the main cause of sex misidentification to be data entry error in the field notebooks, and if so then the proportion of males accidentally registered as females would be about equal to the proportion of females registered as males. However, because survival rates for Steller sea lions are higher for females than males, as is typical for highly sexually dimorphic species, the distribution of errors becomes more skewed with age. There is some debate as to whether correcting these errors introduces bias in the final survival estimates. On the one hand, not correcting sex errors may inflate the survival estimates of adult males, because ”males” that are actually females

will live longer. On the other hand, correcting for these differences may inflate female survival because those females that were incorrectly identified as males and died young are removed from the sample. These potential biases can be significant in particular for cohorts before 2001, where the sex misidentification rate was around 10-15%. We fitted models both for the raw data and data corrected for sex misidentification. In both cases the same models were selected. In our analysis, we quantified the effect of correcting for sexing errors by using two datasets with and without sex correction to estimate survival rates. Estimates of survival without taking correcting for sex misidentification affected estimates of survival for adult males for the older ages, with minimal effects on female survival estimates (see S3 and S4 Figs.). We concluded that if we do not correct errors, the inflation of male survival rates is much greater than the corresponding introduced bias for females when we correct the errors. Thus, our subsequent modeling only used the sex-corrected data. Another source of potential bias would have occurred if we used cohorts that had less than 4 years observation history (from birth year to last year of observation effort). In our analysis we excluded those cohorts, however here we presented differences in survival estimates if we had included cohorts with three or less years of observation. We found a strong bias only in sites with low pup and juvenile resight probabilities (all Kuril Islands). The biggest differences were found in pup survival estimates. Juvenile survival was less affected and by age 4 differences became almost negligible. Including all cohorts on Medny Island and Kozlov Cape did not affect survival estimates, probably due to higher resight rates for the pups and juveniles (see S5 and S6 Figs.).

Resight probability

The best selected models for resight probability included site and time (year) interaction terms. This variation may be related to differences in observation effort. However, all models with any of the effort covariates that we considered (quantified as the number of days that observers spent on each site) had lower weights than the models that included site and time. None of the models including effort were ranked highly, not even making it into the top twenty in the final analysis. The importance of site and time (year) in the variation in resight probability remained, regardless of effort. Therefore, the variability in resight probability was not due to the differences in effort across sites and years. This likely occurred because the vast majority of resights occurred during a limited number of days in late June through early July, and there was actually very little variation in effort during this short window of time, as seen in S7 Fig., showing differences in effort during the summer months between years and sites. The observations on each site and each year were always conducted for at least 4 weeks (between weeks 25 and 28; week 25 begin on June 15). Ninety to Ninety-five percent of marked sea lions were discovered during these weeks each year. Extension of this period provided little additional information on all the Kuril Islands and Kozlov Cape, but on Medny Island a significant number of additional resights occurred between weeks 28 and 32 (S7 Fig.). Even though the total amount of effort did vary between sites and years, it was fairly constant between observation years and always covered the time periods when most annual discoveries occurred. Differences in the terrain on each site were likely responsible for much of the resight probability differences between site. For example, some sites had very rocky topography and the observation blinds were located at a lower height above the rookery while other sites consisted of flat beaches with observation blinds that were over 30 m above the rookery, and these difference likely influence the observers ability to discover new sea lions each year. The project also experienced high observer turnover from year to year and between sites, and over time the observation skills of individuals changed. Resight probability increased with time, but with considerable variability (see S8 Fig.). Additionally, observations on haulouts were important for resights. The

greatest number of resights came from haulouts associated with the Medny Island rookery (Commander Islands), whereas the lowest proportion of resights from haulouts was found for sea lions born on Kozlov Cape and Brat Chirpoev Island (S9 Fig.). Observation effort on haulouts was very limited compared with observations on rookeries. It is likely that differences in effort on haulout sites may have some potential effect on recapture rate, but nonetheless the models that included a covariate of separate effort on haulouts were also not amongst the best models in our selection list.

Basis spline matrix estimation

By generating a B-spline matrix, we were able to reduce the number of parameters needed to estimate age-dependent survival. In our best models four degrees of freedom were chosen because this enabled the model fit which minimized the AIC. Here we provide information on flexibility of those curves using different degrees of freedom. All of the curves, even one with df = 3, provided quite similar results in the Kuril Islands, however models with df > 6 became overparameterized. Comparing differences between curves for Medny Island was difficult, because models with df > 4 became overparameterized. The same was true for Kozlov Cape, where models became overparameterized with df > 5 (see S10 and S11 Figs.).
