# Supplementary figures and images for: Age Specific Survival Rates of Steller Sea Lions at Rookeries with Divergent Population Trends in the Russian Far East
Source: PLoS One. 2015 May 27;10(5):e0127292. doi: 10.1371/journal.pone.0127292 (PMC4446299; doi:10.1371/journal.pone.0127292)

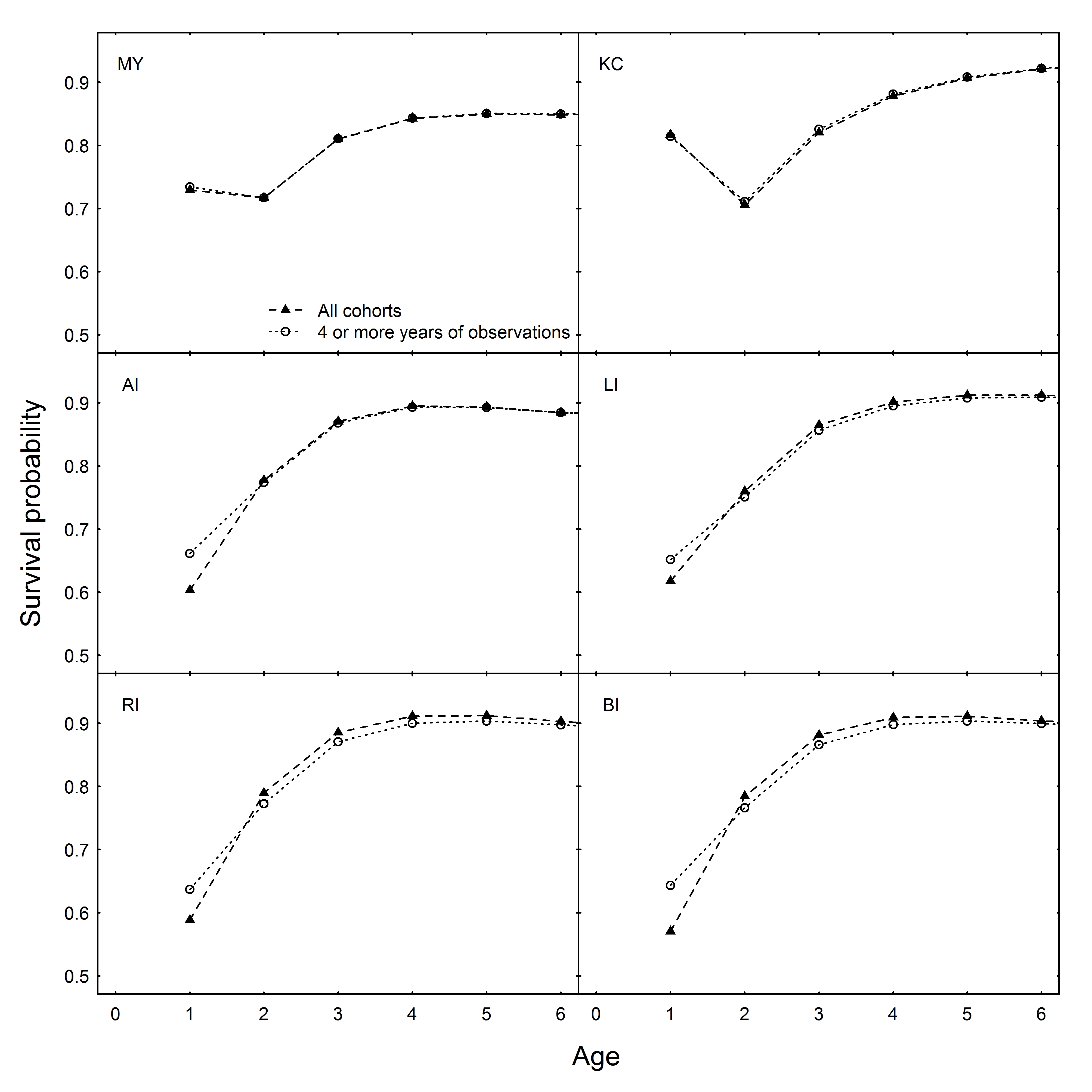

Supplement: S5 Fig — Estimation for Medny Island (MY), Kozlov Cape (KC), Antisferov Island (AI), Lovushki Islands (LI), Raykoke Island (RI), and Brat Chirpoev Island (BI). (TIFF) [file pone.0127292.s006.tiff]

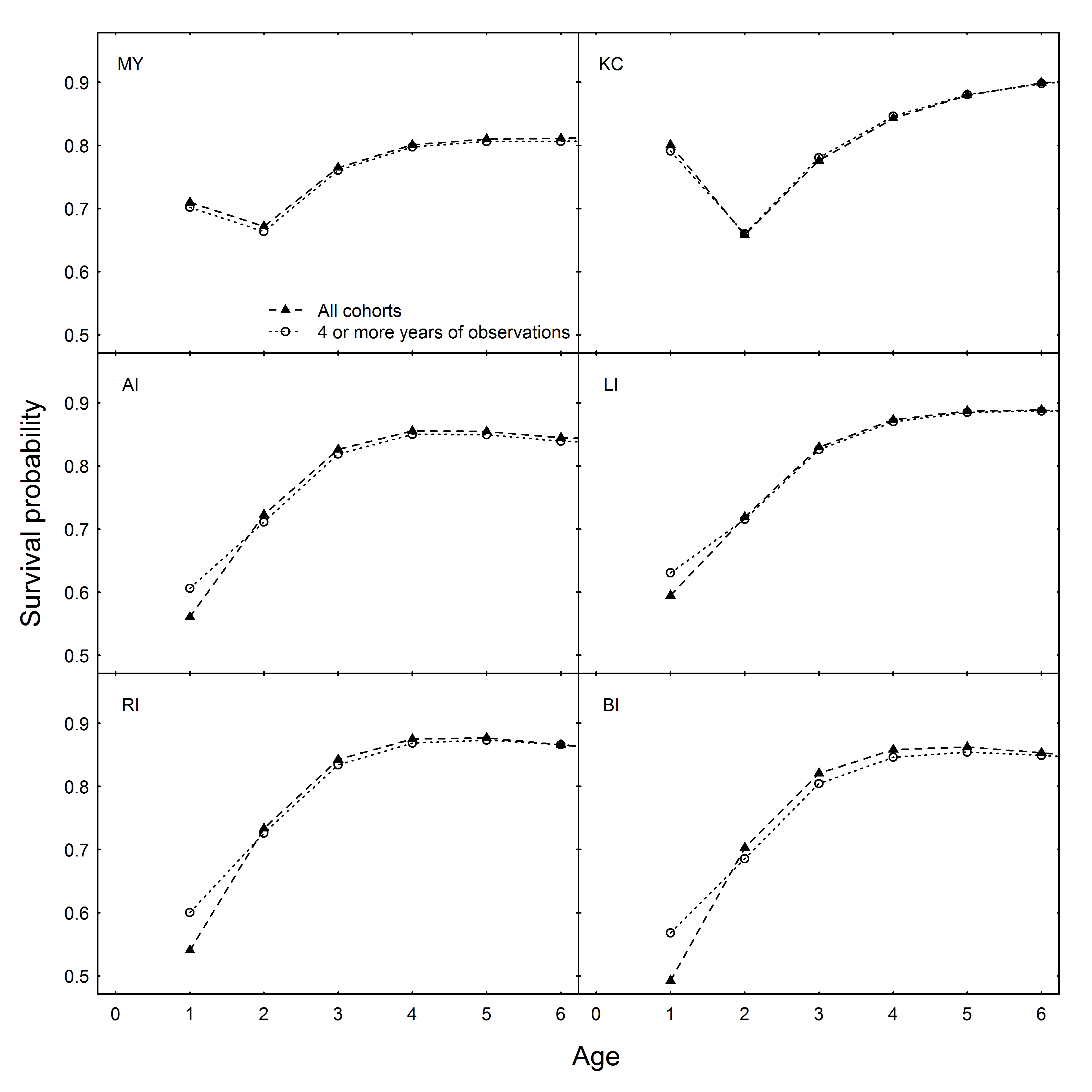

Supplement: S6 Fig — Estimation for Medny Island (MY), Kozlov Cape (KC), Antisferov Island (AI), Lovushki Islands (LI), Raykoke Island (RI), and Brat Chirpoev Island (BI). (TIFF) [file pone.0127292.s007.tiff]

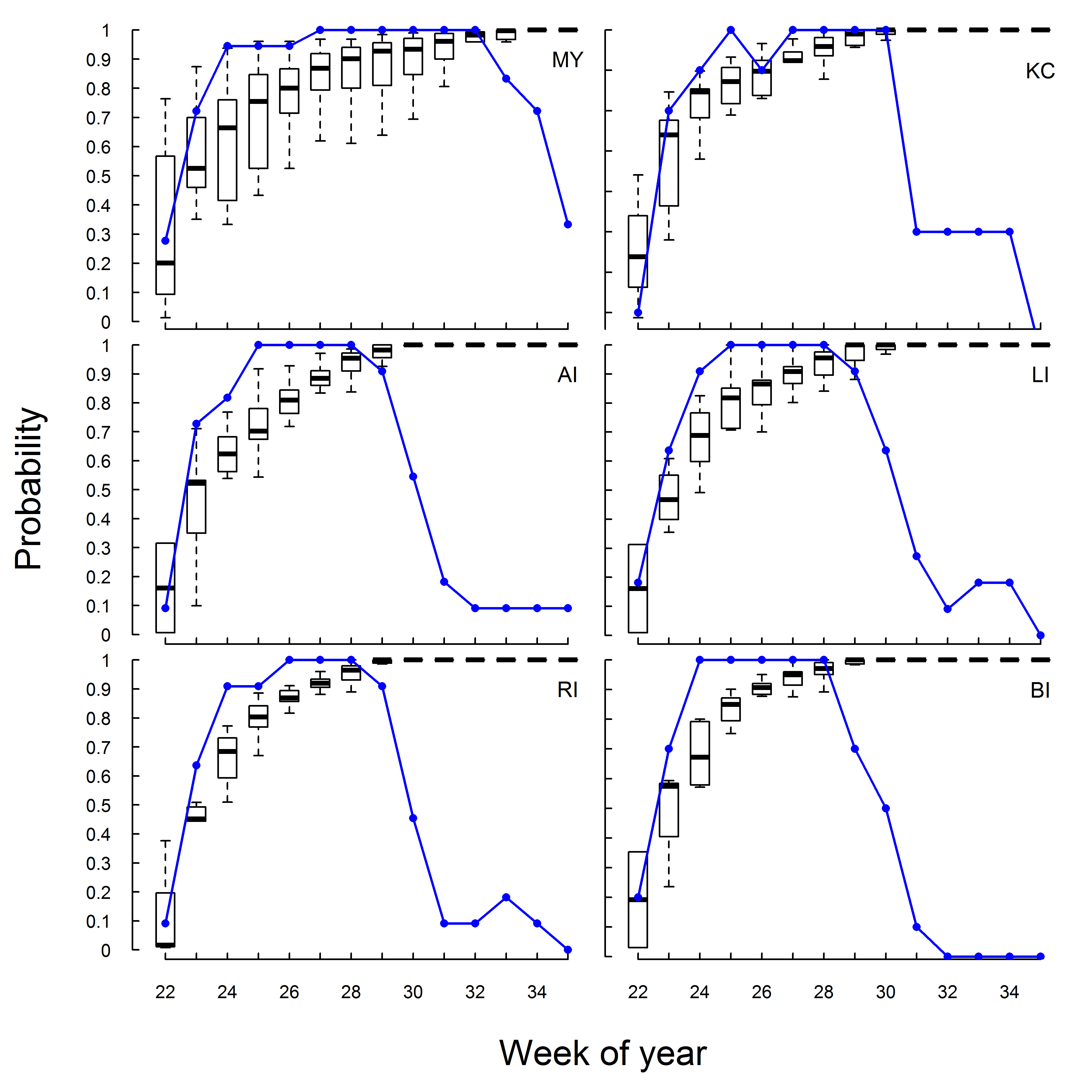

Supplement: S7 Fig — Medny Island (MY), Kozlov Cape (KC), Antisferov Island (AI), Lovushki Islands (LI), Raykoke Island (RI), Brat Chirpoev Island (BI). (TIFF) [file pone.0127292.s008.tiff]

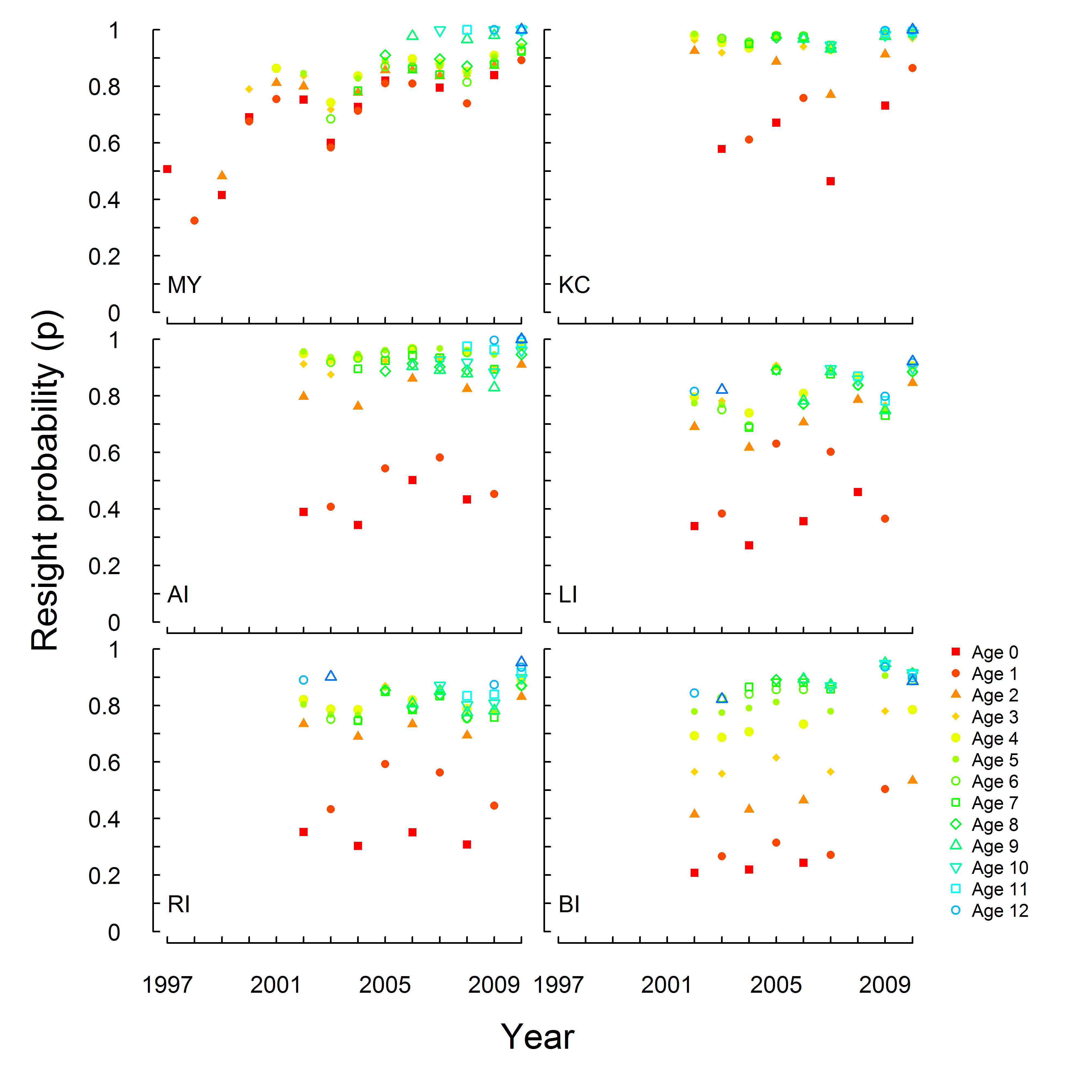

Supplement: S8 Fig — Medny Island (MY), Kozlov Cape (KC), Antisferov Island (AI), Lovushki Islands (LI), Raykoke Island (RI), Brat Chirpoev Island (BI). (TIFF) [file pone.0127292.s009.tiff]

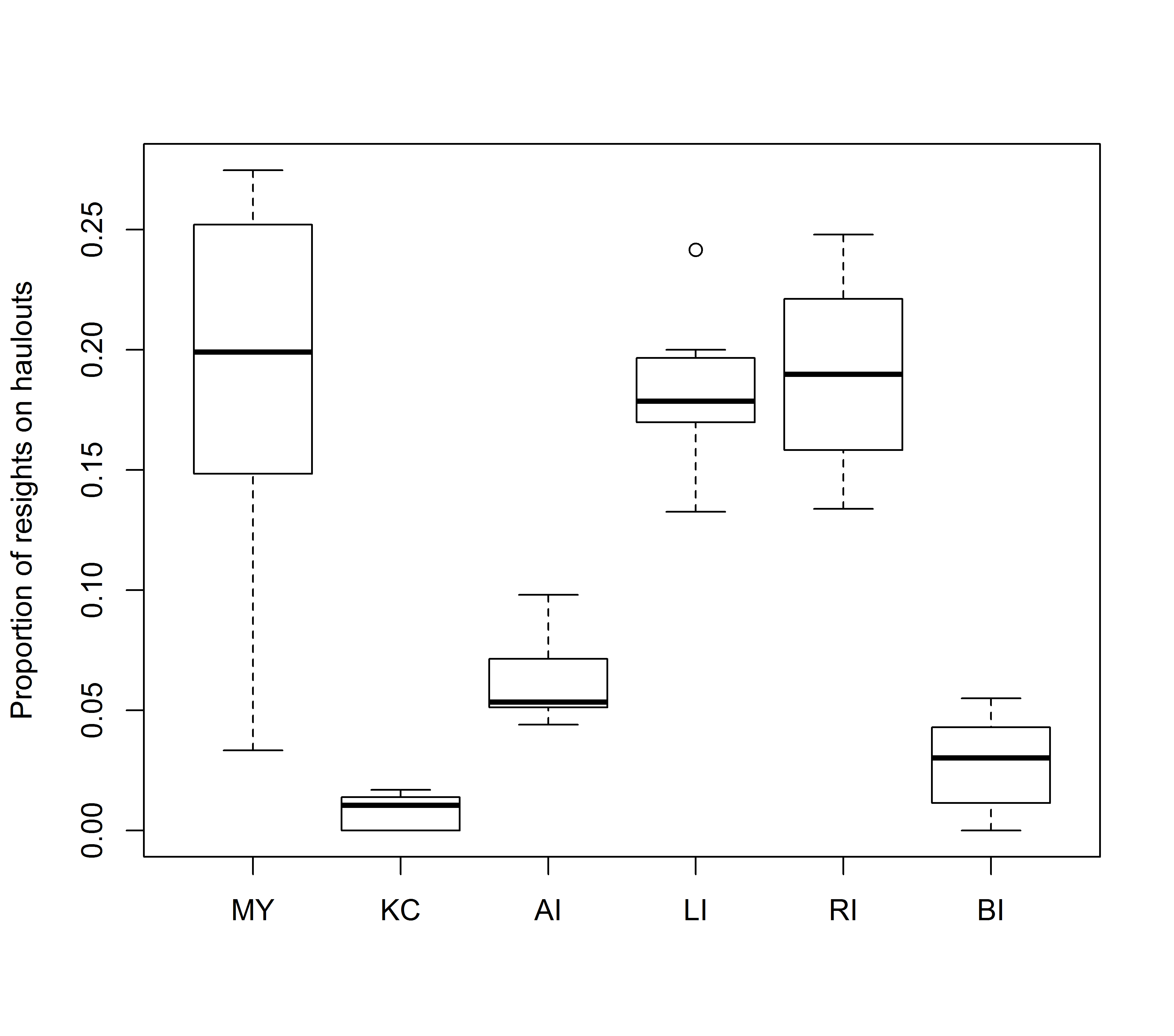

Supplement: S9 Fig — Medny Island (MY), Kozlov Cape (KC), Antisferov Island (AI), Lovushki Islands (LI), Raykoke Island (RI), Brat Chirpoev Island (BI). (TIFF) [file pone.0127292.s010.tiff]

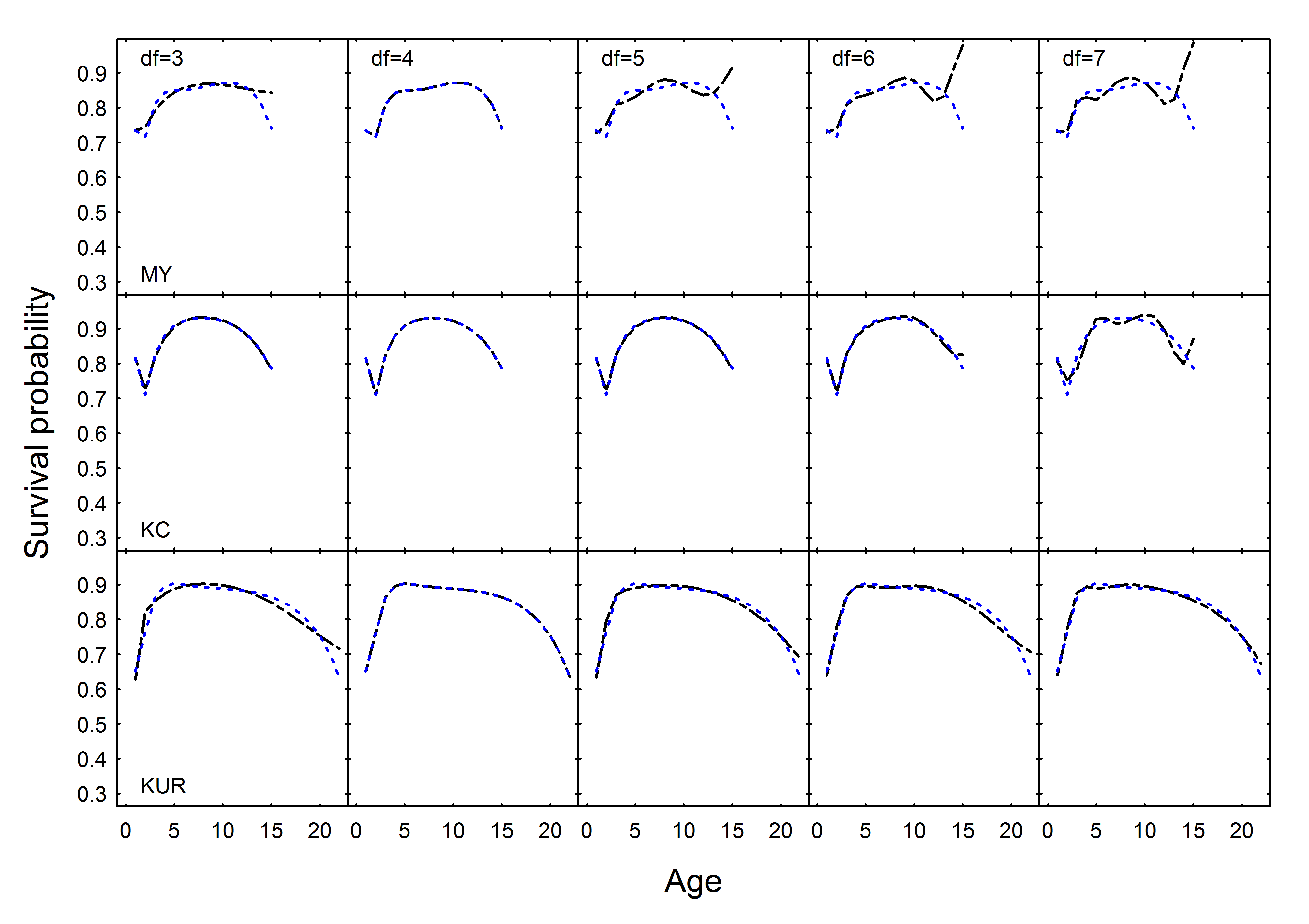

Supplement: S10 Fig — Blue dotted line in each cell represents the best survival model with df = 4. Medny Island (MY), Kozlov Cape (KC), all Kuril Islands (KUR). (TIFF) [file pone.0127292.s011.tiff]

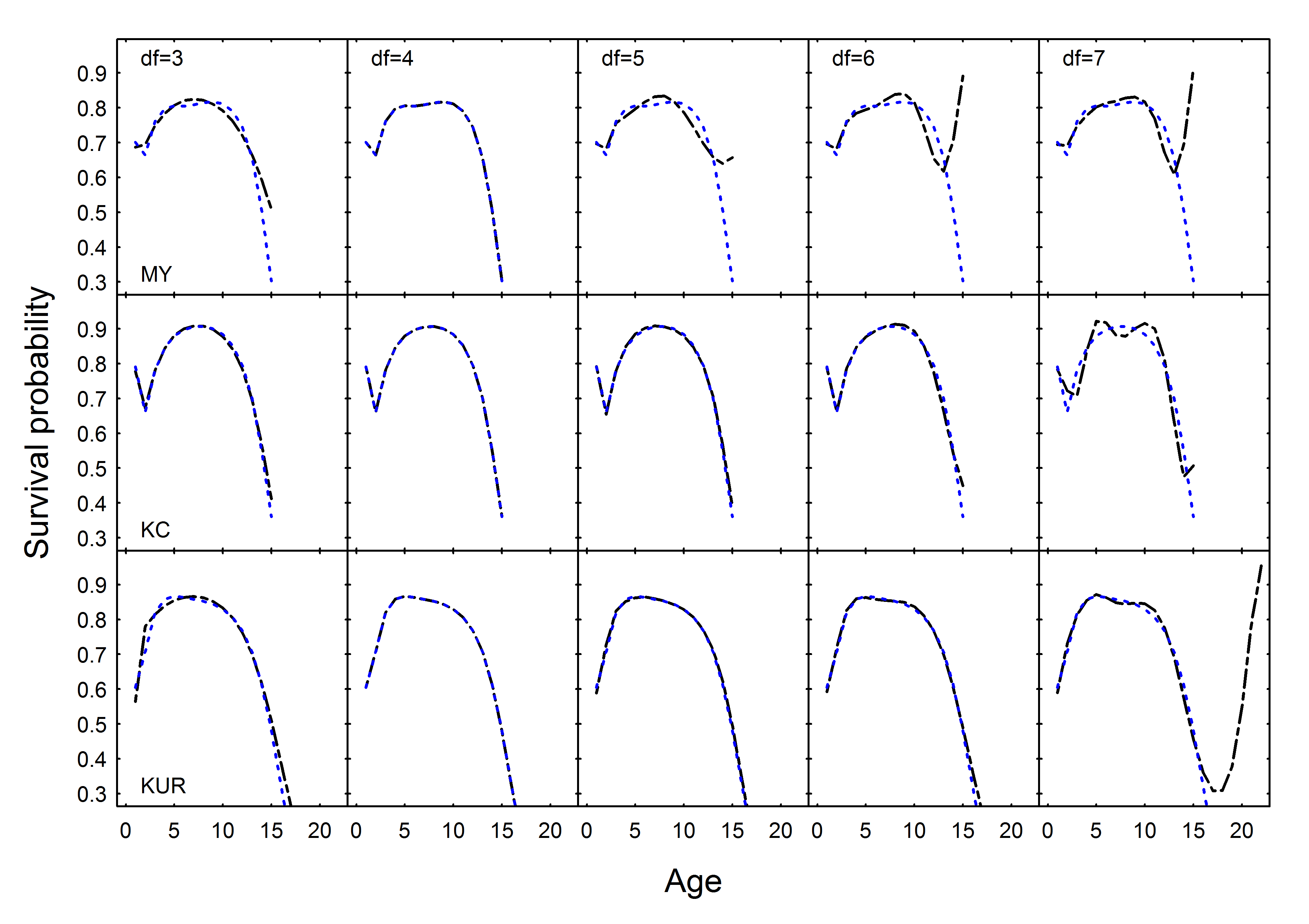

Supplement: S11 Fig — Blue dotted line in each cell represents the best survival model with df = 4. Medny Island (MY), Kozlov Cape (KC), all Kuril Islands (KUR). (TIFF) [file pone.0127292.s012.tiff]
