# Supplementary material for: Age Specific Survival Rates of Steller Sea Lions at Rookeries with Divergent Population Trends in the Russian Far East
Source: PLoS One. 2015 May 27;10(5):e0127292. doi: 10.1371/journal.pone.0127292 (PMC4446299; doi:10.1371/journal.pone.0127292)
Supplement: S1 Table — Estimates based on model 1 in Table 5. (PDF) [file pone.0127292.s013.pdf]

S1 Table

Annual age and sex specific survival and standard error estimations of Steller sea lions branded along the Russian coast during the period 1989-2008, and resighted in the period 1997-2011 (Medny Island) and 2002–2011 (all other rookeries). Estimates based on model 1 in Table 5.

Age	Commander Islands				Eastern Kamchatka				Kuril Islands			
	Females		Males		Females		Males		Females		Males	
	Phi	se	Phi	se	Phi	se	Phi	se	Phi	se	Phi	se
0–1	0.735	0.027	0.701	0.030	0.815	0.051	0.791	0.057	0.651	0.026	0.604	0.026
1–2	0.717	0.019	0.664	0.024	0.711	0.031	0.661	0.036	0.762	0.014	0.707	0.016
2–3	0.810	0.018	0.761	0.022	0.826	0.027	0.781	0.030	0.863	0.012	0.819	0.015
3–4	0.844	0.014	0.797	0.018	0.882	0.018	0.846	0.021	0.896	0.009	0.858	0.011
4–5	0.851	0.016	0.805	0.020	0.909	0.016	0.880	0.020	0.903	0.007	0.867	0.010
5–6	0.851	0.018	0.805	0.024	0.922	0.017	0.897	0.022	0.900	0.007	0.863	0.011
6–7	0.854	0.018	0.809	0.025	0.929	0.016	0.906	0.021	0.896	0.008	0.858	0.012
7–8	0.860	0.019	0.815	0.025	0.931	0.015	0.907	0.020	0.893	0.009	0.852	0.012
8–9	0.867	0.023	0.817	0.030	0.929	0.016	0.900	0.023	0.891	0.009	0.843	0.013
9–10	0.872	0.027	0.811	0.038	0.922	0.019	0.884	0.029	0.888	0.009	0.829	0.017
10–11	0.872	0.031	0.791	0.047	0.911	0.025	0.852	0.040	0.886	0.009	0.806	0.021
11–12	0.864	0.038	0.744	0.063	0.894	0.030	0.795	0.053	0.882	0.010	0.768	0.027
12–13	0.846	0.059	0.656	0.106	0.869	0.036	0.700	0.071	0.878	0.012	0.707	0.035
13–14	0.809	0.117	0.506	0.196	0.834	0.061	0.551	0.117	0.872	0.015	0.613	0.055
14–15	0.741	0.241	0.303	0.275	0.787	0.130	0.361	0.190	0.864	0.018	0.481	0.095
0-15	0.055		0.005		0.121		0.017		0.102		0.015	
15–16									0.85	0.02	0.32	0.14
16–17									0.84	0.02	0.17	0.14
17–18									0.82	0.03	0.07	0.09
18–19									0.79	0.03	0.02	0.05
19–20									0.75	0.05	0.01	0.02
20–21									0.70	0.07	0.00	0.00
21–22									0.64	0.11	0.00	0.00
0-22									0.016		0.000	
