# Supplementary material for: Age Specific Survival Rates of Steller Sea Lions at Rookeries with Divergent Population Trends in the Russian Far East
Source: PLoS One. 2015 May 27;10(5):e0127292. doi: 10.1371/journal.pone.0127292 (PMC4446299; doi:10.1371/journal.pone.0127292)
Supplement: S2 Table — Estimates based on model 1 in Table 5. (PDF) [file pone.0127292.s014.pdf]

S2 Table

Cumulative age and sex specific survival with 95% confidence intervals of Steller sea lions branded along the Russian coast during the period 1989-2008, and resighted in the period 1997-2011 (Medny Island) and 2002–2011 (all other rookeries). Estimates based on model 1 in Table 5.

	Commander Islands						Eastern Kamchatka						Kuril Islands					
	Females			Males			Females			Males			Females			Males		
Age	Surv.	lcl	ucl	Surv.	lcl	ucl	Surv.	lcl	ucl	Surv.	lcl	ucl	Surv.	lcl	ucl	Surv.	lcl	ucl
0	1	1	1	1	1	1	1	1	1	1	1	1	1	1	1	1	1	1
1	0.735	0.681	0.787	0.701	0.645	0.758	0.815	0.717	0.909	0.791	0.691	0.899	0.651	0.605	0.702	0.604	0.556	0.657
2	0.526	0.479	0.571	0.466	0.418	0.517	0.579	0.498	0.666	0.522	0.430	0.615	0.496	0.454	0.540	0.428	0.389	0.470
3	0.426	0.385	0.468	0.355	0.312	0.399	0.478	0.407	0.562	0.408	0.334	0.488	0.428	0.390	0.466	0.350	0.316	0.387
4	0.360	0.322	0.396	0.283	0.247	0.323	0.422	0.359	0.496	0.345	0.279	0.413	0.384	0.350	0.419	0.301	0.271	0.334
5	0.306	0.273	0.339	0.228	0.197	0.261	0.383	0.324	0.450	0.304	0.245	0.365	0.347	0.316	0.378	0.261	0.234	0.291
6	0.260	0.231	0.291	0.183	0.158	0.212	0.353	0.300	0.417	0.273	0.221	0.331	0.312	0.283	0.342	0.225	0.201	0.251
7	0.222	0.196	0.249	0.148	0.126	0.174	0.328	0.277	0.385	0.247	0.199	0.300	0.280	0.252	0.307	0.193	0.172	0.217
8	0.191	0.167	0.216	0.121	0.100	0.144	0.306	0.256	0.363	0.224	0.178	0.272	0.250	0.226	0.276	0.164	0.145	0.185
9	0.166	0.145	0.190	0.099	0.082	0.120	0.284	0.234	0.335	0.202	0.159	0.248	0.222	0.201	0.246	0.139	0.122	0.157
10	0.144	0.124	0.166	0.080	0.066	0.098	0.262	0.217	0.310	0.178	0.141	0.223	0.198	0.178	0.219	0.115	0.101	0.131
11	0.126	0.107	0.148	0.063	0.050	0.080	0.238	0.196	0.284	0.152	0.118	0.190	0.175	0.158	0.194	0.093	0.080	0.107
12	0.109	0.091	0.130	0.047	0.034	0.063	0.213	0.174	0.260	0.121	0.091	0.154	0.154	0.139	0.172	0.071	0.061	0.083
13	0.092	0.073	0.114	0.031	0.019	0.047	0.185	0.149	0.228	0.084	0.059	0.118	0.136	0.121	0.152	0.050	0.042	0.060
14	0.074	0.050	0.103	0.016	0.004	0.031	0.154	0.118	0.198	0.047	0.024	0.074	0.118	0.105	0.133	0.031	0.023	0.040
15	0.055	0.017	0.101	0.005	0.000	0.019	0.121	0.074	0.174	0.017	0.003	0.042	0.102	0.090	0.115	0.015	0.009	0.022
16													0.087	0.076	0.099	0.005	0.001	0.010
17													0.073	0.063	0.084	0.001	0.000	0.003
18													0.060	0.051	0.070	0.000	0.000	0.000
19													0.047	0.040	0.056	0.000	0.000	0.000
20													0.035	0.029	0.044	0.000	0.000	0.000
21													0.025	0.018	0.033	0.000	0.000	0.000
22													0.016	0.010	0.024	0.000	0.000	0.000

Note that confidence intervals estimated using bootstrap approach. Surv. = cumulative survival, lcl = lower confidence level, ucl = upper confidence level.
